# Glutaminolysis dynamics during astrocytoma progression correlates with tumor aggressiveness

**DOI:** 10.1186/s40170-021-00255-8

**Published:** 2021-04-28

**Authors:** Yollanda E. Moreira Franco, Maria Jose Alves, Miyuki Uno, Isabele Fattori Moretti, Marina Trombetta-Lima, Suzana de Siqueira Santos, Ancely Ferreira dos Santos, Gabriel Santos Arini, Mauricio S. Baptista, Antonio Marcondes Lerario, Sueli Mieko Oba-Shinjo, Suely Kazue Nagahashi Marie

**Affiliations:** 1 Laboratory of Molecular and Cellular Biology (LIM 15), Department of Neurology, Faculdade de Medicina FMUSP, Universidade de São Paulo, São Paulo, 01246–903 Brazil; 2 Department of Molecular Pharmacology, University of Groningen, 9713 Av, Groningen, The Netherlands; 3 School of Applied Mathematics, Fundação Getulio Vargas, Rio de Janeiro, 22250-900 Brazil; 4 Institute of Chemistry, Department of Biochemistry, Universidade de São Paulo, CEP, São Paulo, 05508-000 Brazil; 5 Department of Internal Medicine, Division of Metabolism, Endocrinology, and Diabetes, University of Michigan, Ann Arbor, Michigan USA

**Keywords:** Glutaminolysis, GBM, Low-grade astrocytoma, IDH1 mutation, Astrocytoma progression

## Abstract

**Background:**

Glioblastoma is the most frequent and high-grade adult malignant central nervous system tumor. The prognosis is still poor despite the use of combined therapy involving maximal surgical resection, radiotherapy, and chemotherapy. Metabolic reprogramming currently is recognized as one of the hallmarks of cancer. Glutamine metabolism through glutaminolysis has been associated with tumor cell maintenance and survival, and with antioxidative stress through glutathione (GSH) synthesis.

**Methods:**

In the present study, we analyzed the glutaminolysis-related gene expression levels in our cohort of 153 astrocytomas of different malignant grades and 22 non-neoplastic brain samples through qRT-PCR. Additionally, we investigated the protein expression profile of the key regulator of glutaminolysis (GLS), glutamate dehydrogenase (GLUD1), and glutamate pyruvate transaminase (GPT2) in these samples. We also investigated the glutathione synthase (GS) protein profile and the GSH levels in different grades of astrocytomas. The differential gene expressions were validated in silico on the TCGA database.

**Results:**

We found an increase of glutaminase isoform 2 gene (*GLSiso2*) expression in all grades of astrocytoma compared to non-neoplastic brain tissue, with a gradual expression increment in parallel to malignancy. Genes coding for *GLUD1* and *GPT2* expression levels varied according to the grade of malignancy, being downregulated in glioblastoma, and upregulated in lower grades of astrocytoma (AGII–AGIII). Significant low GLUD1 and GPT2 protein levels were observed in the mesenchymal subtype of GBM.

**Conclusions:**

In glioblastoma, particularly in the mesenchymal subtype, the downregulation of both genes and proteins (GLUD1 and GPT2) increases the source of glutamate for GSH synthesis and enhances tumor cell fitness due to increased antioxidative capacity. In contrast, in lower-grade astrocytoma, mainly in those harboring the *IDH1* mutation, the gene expression profile indicates that tumor cells might be sensitized to oxidative stress due to reduced GSH synthesis. The measurement of *GLUD1* and *GPT2* metabolic substrates, ammonia, and alanine, by noninvasive MR spectroscopy, may potentially allow the identification of *IDH1*^mut^ AGII and AGIII progression towards secondary GBM.

**Supplementary Information:**

The online version contains supplementary material available at 10.1186/s40170-021-00255-8.

## Background

Cancer is among the leading causes of death worldwide [1]. Although the tumors of the central nervous system (CNS) are less frequent, representing about 3% of all tumors, they are among the most aggressive [2, 3]. Gliomas, which originate from glial cells or their precursors, represent more than 80% of primary brain tumors [4–6]. Glioblastoma (GBM), the most frequent adult malignant glioma and classified as a WHO grade IV astrocytoma, has been stratified according to the molecular profile as proneural, classical, and mesenchymal subtypes [7, 8], which partially predict the clinical outcome. The proneural subtype characterized by the presence of *IDH* mutation has been associated with a better prognosis [9], while the mesenchymal subtype with *NF1* or *RB1* mutations has presented the worst outcome, with an average overall survival of 8–11 months [10]. More recently, an impact of the mutational landscape on the response to immunotherapy and on the acquired resistance to temozolomide (TMZ) has been demonstrated in gliomas [11].

The capacity of cancer cells to reprogram their metabolisms to support rapid proliferation is another cancer hallmark with prognostic impact [12, 13]. Interestingly, metabolic enzymes with high catalytic activity are found upregulated in different kinds of tumor and are associated with poor survival [14]. Glutamine (Gln) metabolism is upregulated by various oncogenic signaling pathways [15] and is relevant in cancer development due to its involvement in mTOR signaling, autophagy, and antioxidative stress and as a source of glutathione (GSH) and for anaplerosis [12, 13, 16]. Moreover, Gln uptake and the rate of glutaminolysis are known to be related to tumor growth [17–19]. Besides, we previously have observed that Gln transporters are upregulated in astrocytoma [20]. The dependence of cancer cells on Gln makes glutaminolysis an attractive cancer therapy target [15, 21–23]. Gln is a non-essential amino acid, consumed largely by proliferating cancer cells in vitro, which are often dependent on extracellular Gln for survival [24]. Gln carbon contributes to aspartate, glutamate (Glu), and tricarboxylic acid (TCA) cycle metabolites via glutaminolysis [15]. High rates of glutaminolysis support rapid proliferation by supplying precursors to low-flux biosynthetic pathways [24]. Current attempts to target glutaminolysis clinically have focused largely on inhibiting glutaminase. Chemical inhibitors have been found to decrease cancer cell proliferation in both in vitro/in vivo models [25–27].

The metabolic ending of glutamine–derived Glu is, apart from α–ketoglutarate (α–KG), lactate and GSH being an important nitrogen donor for cell growth and proliferation [28, 29]. Additionally, studies about *IDH* mutation showed significantly reduced levels of Gln and Glu levels were, which implies replenishment of α–KG by glutaminolysis. Consequently, wild-type (wt) gliomas presented high levels of intracellular Glu, which is released via the Gln/cysteine antiporter System X_C_^–^ in exchange for cysteine. GSH is considered a potent antioxidant and the main factor responsible for treatment resistance in gliomas or other neoplastic cells [30, 31]. Therapeutic attempts have been aimed at GSH depletion by inhibiting the X_C_^–^ transporter [32], which is responsible for counter–transport of Glu and cysteine–a substrate–limiting GSH synthesis [30, 31]. This exchange is favorable for the cancer cells because Cys is a major component of the antioxidant GSH, which in turn is an antagonist of reactive oxygen species (ROS) [28].

In the present study, we analyzed the expression profile of the genes related to glutaminolysis in different grades of astrocytomas and, more specifically, in the molecular subtypes of GBM, and lower malignant grades of astrocytoma regarding *IDH1* mutation status. We searched for differential features of glutaminolysis related to GBM aggressiveness and malignant progression of low-grade astrocytomas with *IDH1* mutation, which may help to better characterize the metabolic features associated with GBM aggressiveness and to tumor malignant progression.

## Methods

### Tissue sample and ethical statement

Samples were snap-frozen in liquid nitrogen immediately following surgical removal and macro dissected before RNA extraction. A 4-μm-thick cryosection of each sample was stained with hematoxylin-eosin and analyzed under a light microscope for assessment of necrotic, cellular debris, and NN areas (in tumoral samples). For gene expression, we analyzed 153 human astrocytoma samples stratified according to the WHO classification (2007) [33] as 23 astrocytomas grade I (AGI), 26 astrocytomas grade II (AGII), 18 astrocytomas grade III (AGIII), and 86 GBM. Non-neoplastic brain samples (NN) were used as control (22 cases). For the GLS protein analysis, NN (5), AGI (4), AGII (4), AGIII (2), and GBM (6) were evaluated. For GLUD1 and GPT2 protein analysis, we explored AGII-*IDH*^wt^ (4), AGII-*IDH*^mut^ (6), GBM of mesenchymal subtype (GBM-MS) (7), and GBM of proneural subtype (GBM-PN) (5) samples. For glutathione synthetase (GS) protein analysis, we explored AGII-*IDH*^wt^ (4), AGII-*IDH*^mut^ (4), GBM-MS (4), and GBM-PN (4). Tumor samples were obtained from surgery of patients treated by the Neurosurgery Group of Department of Neurology at Hospital das Clinicas at the School of Medicine of University of São Paulo, from 2000 to 2007. NN brain tissue samples were collected from epilepsy patients subjected to temporal lobectomy.

### Cell culture

The U87MG cell line was acquired from ATCC and authenticated by short tandem repeats (STR) analysis using GenePrint 10 System (Promega, Madison, WI). Cells were cultured in monolayer with DMEM medium (Dulbecco’s modified Eagle’s medium, Thermo Fisher Scientific, Carlsbad, CA), 10% fetal bovine serum, and 100 μg/ml streptomycin and 100 IU/ml penicillin.

### RNA extraction and cDNA synthesis

Total RNA was extracted from frozen tissues (tumor and NN) using the RNeasy Mini Kit (Qiagen, Hilden, Germany) following the manufacturer’s instructions. The RNA concentration and purity were evaluated by NanoDrop, and ratios of 260/280 measures ranging from 1.8 to 2.0 were considered satisfactory for purity standards. RNA quality was checked by electrophoresis in agarose gel. A conventional reverse transcription reaction was performed to yield single-stranded cDNA. The first strand of cDNA was synthesized from 1 μg of total RNA previously treated with 1 unit of DNase I (FPLC–pure, GE Healthcare, Uppsala, Sweden) using random and oligo (dT) primers, RNase inhibitor, and SuperScript III reverse transcriptase according to the manufacturer’s recommendations (Thermo Fisher Scientific). The resulting cDNA was subsequently treated with 1 unit of RNase H (GE Healthcare), diluted with TE buffer, and stored at – 20 °C until later use.

### Analysis of gene expression by quantitative real-time PCR (qRT–PCR)

The relative expression levels of genes involved in the glutaminolysis pathway *GLS, GLSiso1, GLSiso2, GLS2, GLUD1, GOT1, GOT2,* and *GPT2* were analyzed by qRT–PCR, using the SYBR Green approach. The expression of *ASCT2* and *LAT1* genes were previously described by our group [20]. A geometric mean of three suitable reference genes was used for normalizing the quantitative data: hypoxanthine phosphoribosyltransferase (*HPRT*), glucuronidase beta (*GUSβ*), and TATA box binding protein (*TBP*) [34]. The primers were designed to amplify 80–120 bp amplicons, with a melting temperature of 60 °C and were synthesized by IDT (Integrated DNA Technologies, Coralville, IA). The primers information is described in Table 1.

**Table 1:** The primer sequences and the concentration used in qRT–PCR

Genes	Forward primer (5′–3′)	Reverse Primer (5′–3′)	Concentration (nM)
*GLS*	CAGGGCAGTTTGCTTTCCAT	GAGACCAGCACATCATACCCAT	200
*GLSiso1*	GCAGAGGGTCATGTTGAAGTTGT	GGTGTCCAAAGTGCAGTGCTT	200
*GLSiso2*	ATCCTCGAAGAGAAGGTGGTGA	GCAAGTTCTTGTTGGAGACTTTCA	400
*GLS2*	ATCCTCGAAGAGAAGGTGGTGA	ATGGCTGACAAGGCAAACCT	200
*GLUD1*	TGGCATACACAATGGAGCGT	TCTCAATGGCATTAACATAGGCA	400
*GOT1*	CTGTGCCCAGTCCTTCTCCA	GATGCTCTCAGGTTCTTTTCCAA	400
*GOT2*	CTTGAGGTTGGAGACCAGTTGAGT	GATTGCTGCTGCCATTCTGA	400
*GPT2*	GGCTTTGGGCAGAGGGAA	TCACGCGTACTTCTCCAGGAA	200
*HPRT*	TGAGGATTTGGAAAGGGTGT	GAGCACACAGAGGGCTACAA	200
*GUSβ*	AAATACGTGGTTGGAGAGCTCATT	CCGAGTGAAGATCCCCTTTTTA	400
*TBP*	AGGATAAGAGAGCCACGAACCA	CTTGCTGCCAGTCTGGACTGT	200

To ensure the efficiency of amplification and analysis of melting curves, which gave a single peak for all PCR products, standard curves with varying concentrations of the primer pairs of each gene were performed. The optimal primer concentration was determined as the lower concentration which did not affect the cycle threshold (Ct) and displayed the maximum amplification efficiency while minimizing non-specific amplification. Additionally, the amplified PCR product sizes were checked by agarose gel electrophoresis. The SYBR Green I amplification mixtures (12 μl) were composed of cDNA, Power SYBR Green I Master Mix (Thermo Fisher Scientific), and the reverse and forward primers. The qRT–PCR was done in duplicate using the ABI Prism 7500 (Thermo Fisher Scientific) as follows: 2 min at 50 °C, 10 min of polymerase activation at 95 °C, and 40 cycles of 15 s at 95 °C and 1 min at 60 °C. The following equation was applied to calculate gene expression in tumor and NN tissue samples: 2^–ΔCt^, where ΔCt = Ct of a specific gene–geometric mean Ct of housekeeping genes [35].

### Analysis of protein expression by western blotting

The samples and U87MG cells were homogenized with RIPA lysis buffer (50 mM Tris–HCl, 1% NP–40, 0.25% Na deoxycholate, 150 mM NaCl, 1 mM EDTA) supplemented with a cocktail of protease inhibitors (Sigma–Aldrich, St Louis, MO). The protein concentration was determined using the Bradford reagent. All samples (20 μg protein) were resolved by electrophoresis on 4–12% gradient gels in SDS–PAGE using electrophorese buffer NuPAGE MOPS SDS (Thermo Fisher Scientific) and transferred onto PVDF membrane by iBlot Dry Blotting System (Thermo Fisher Scientific). Then the remaining binding sites of the membranes were blocked with skimmed milk powder solution at 5% diluted in Tris-buffered saline and 0.1%Tween 20 (TBST). Subsequently, the membranes were incubated overnight with the primary antibody, anti–GLS (1:1,000), anti-GS (1:2,000) from Abcam (Cambridge, MA), and anti-GLUD1, anti-GPT2 (1:1,000) from Thermo Fisher and then diluted in TBST with 5% bovine serum albumin (BSA) solution. β–actin (1:5.000) (Sigma–Aldrich) was used as a loading control. The membranes were incubated with peroxidase-conjugated secondary antibody anti-rabbit and anti-mouse (1:5.000) (Sigma–Aldrich), also diluted in TBST 5% BSA. The protein levels were detected using the chemiluminescence detection method (Western Lightning Plus–ECL, Enhanced Chemiluminescence Substrate, Perkin Elmer, Waltham, MA). The detection of the chemiluminescent signal was performed in the Photo QuantLAS 4000 mini (GE Healthcare) photo documentation system and the bands were analyzed and quantified using ImageJ software (obtained from imagej.nih.gov/ij/download/).

### GSH measurement

Tissue samples were resuspended in PSB–0.5% NP40 (pH 6) and homogenized in syringes with an insulin needle 10 times. An aliquot of each sample was separated for protein quantification. Eighty microliters were processed with 250 μL of cold GSH extraction buffer (KClO_4_ 50 mM; EDTA 10 mM; H_3_PO_4_ 0.1% (v/v), pH 5) and 40 μL of cold metaphosphoric acid 5% (v/v). The samples were vortexed for 1 min and centrifuged at 8000×*g* (10 min, 4 °C). The supernatants were used as 1:10 dilutions. GSH was measured using a fluorometric detection assay kit (ab138881, Abcam) assay according to the manufacturer’s instructions. This assay is based on the fluorescent properties of thiol green, which is a non-fluorescent dye that becomes strongly fluorescent upon reacting directly with GSH. The fluorescence intensity was evaluated at an excitation wavelength of 490 nm and an emission wavelength of 520 nm using a 96-well plate in a spectrofluorometer (SpectraMAX M2, Molecular Devices, Sunnyvale, CA). GSH concentration was calculated by interpolation of a standard curve and results were expressed as pmol/μg of total protein.

### TCGA data analysis

The gene expression from the RNAseq GBM dataset was downloaded (Genomics Data Commons Data Portal—https://portal.gdc.cancer.gov/) and normalized by DEseq R software. Normalized read counts were converted to a *z*-score for heat map visualization.

### Statistical analysis

Statistical analysis was conducted with SPSS for Windows, version 20.0 (IBM Corporation, Armonk, NY), and GraphPad Prism (version 5.02, San Diego, CA). Comparisons were considered statistically significant when *p* < 0.05. The non-parametric Kruskal-Wallis and post hoc Dunn tests were used to analyze the differences in mRNA relative expression in different grades of astrocytomas. The correlation analysis between gene expression values was assessed by the non-parametric Spearman-rho correlation test. The variation of specificity and sensibility of gene expression levels was analyzed using the receiver operating characteristic (ROC) curve. Among the continuous variables categorized through the ROC curve, the value with the best sensitivity and specificity was chosen as the cut-off value. The area under the ROC curve (AUC) was used to measure how the expression levels could distinguish between two groups. The gene expressions were classified as hyper or hypoexpressed based on this cut-off value. The comparison of protein expression analysis was carried by two-way ANOVA and Bonferroni post-test.

## Results

### *GAC (GLSiso2*) expression increases in parallel to astrocytoma malignancy

**Fig. 1:**
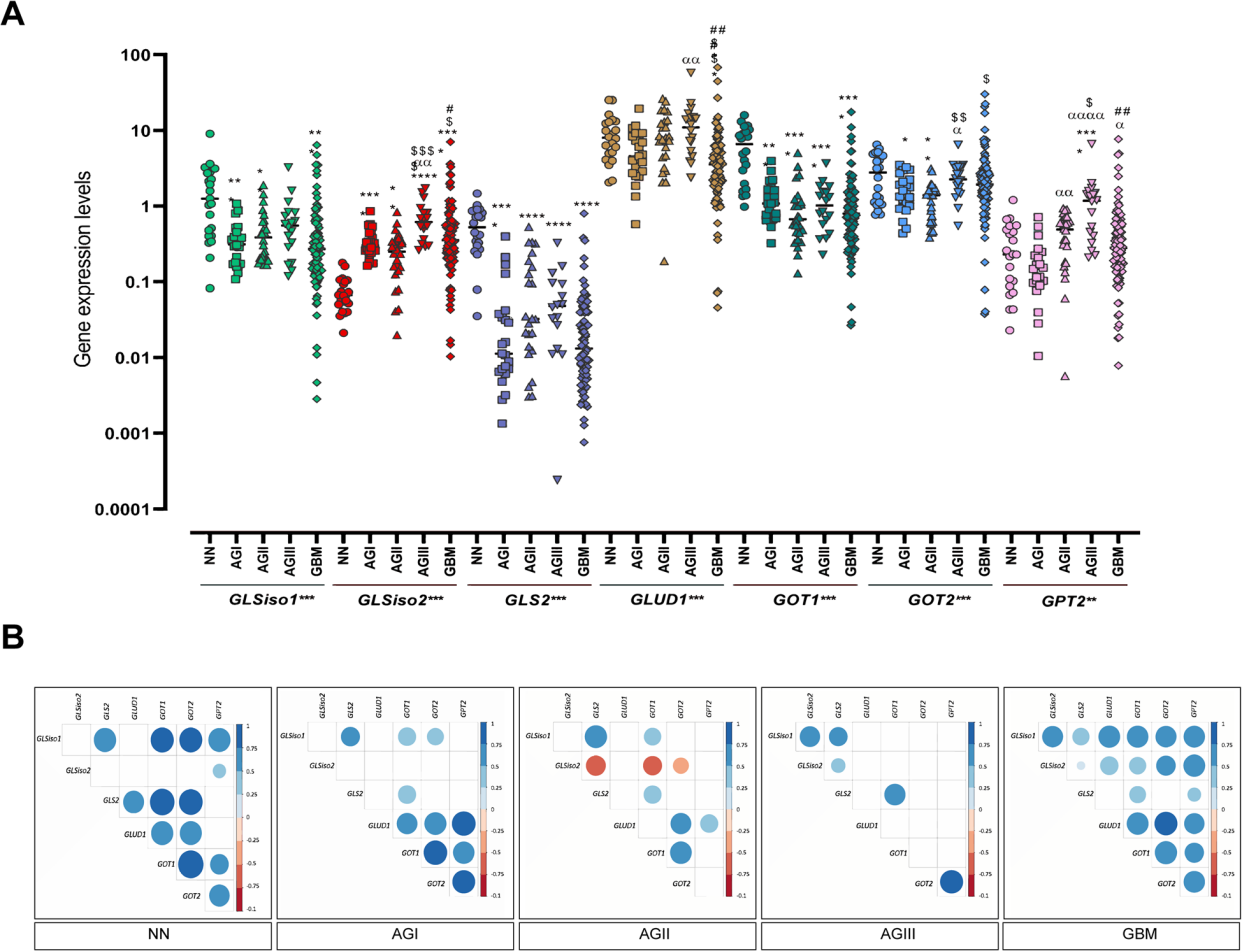
Expression analysis of genes coding for glutaminolysis in astrocytomas of different malignant grades. **a** *GLSiso1, GLSiso2, GLS, GLS2, GLUD1, GOT1, GOT2,* and *GPT2* expression levels in non-neoplastic brain tissue (NN) compared to pilocytic astrocytoma (AGI), low-grade astrocytoma (AGII), anaplastic astrocytoma (AGIII), and glioblastoma (GBM). The expression levels differ significantly among the groups for all genes analyzed (**p = 0.002, ***p < 0.0001, Kruskal-Wallis test) and between NN and each tumor group (post hoc Dunn test, where ****p < 0.0001, ***p < 0.0005, **p < 0.005, and *p < 0.05. The significant comparison between the groups is represented by different symbols: NN (*), AGI (α), AGII ($), and AGIII (#). Horizontal bars show the median of each group. The results are presented on a log10 scale. **b** Correlation matrix showing the gene expression correlations with each other in all groups analyzed. Positive correlations are shown in blue and negative correlations in dark orange. The color intensiveness and the size of the circle are proportional to the value of *r* by the Spearman test. Only the correlations with *p* < 0.05 are plotted

**Fig. 2:**
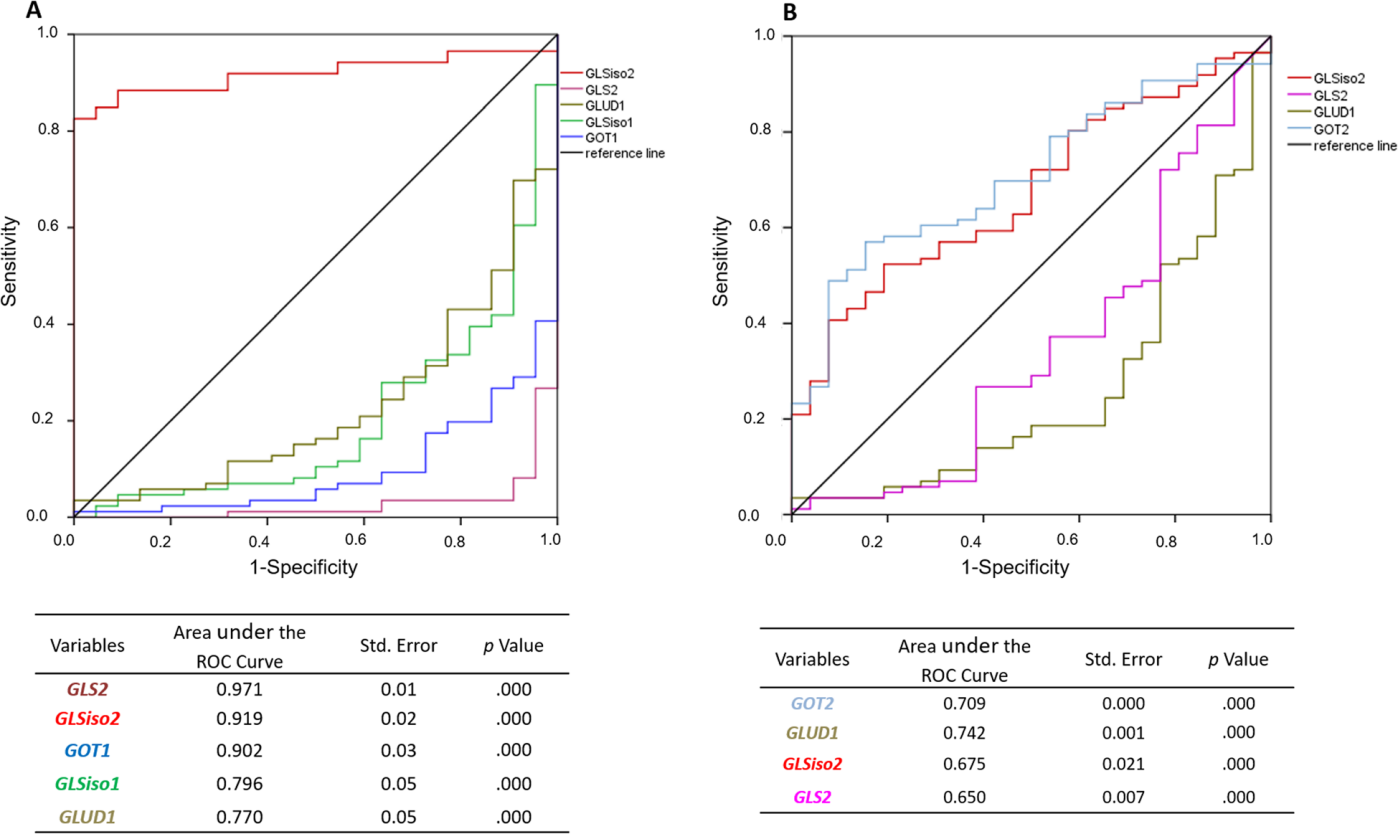
ROC curves for glutaminolysis pathway gene expressions. ROC curves for *GLSiso1*, *GLSiso2*, *GLS2*, *GLUD1,* and *GOT1* and *GOT2* expression levels, showing sensitivity and specificity of gene expression. The AUC values represent the accuracy of the individual gene for distinguishing between GBM and non–neoplastic tissue in **a**, and between GBM and AGII samples in **b**

Once Gln is transported into cells by ASCT2 (SLC1A5) and LAT1 (SLC7A5), it is converted to Glu by glutaminases (GLS and GLS2). This is a critical step, as Glu does not efficiently cross the blood-brain barrier, and brain interstitial Glu concentration is maintained essentially through its synthesis [36–38]. *GLS* presents two isoforms: *KGA* (*GLSiso1,* cytosolic) and *GAC* (*GLSiso2*, mitochondrial). The *GLS*iso2 is derived from an alternative exon splicing at the 3′–end terminal, excluding the ankyrin repeats at the C–terminus coded by the last four exons of the *GLSiso1* transcript. Thus, the *GLSiso2* is shorter than the *GLSiso1*, with a distinct C–terminal [39]. Expression analysis of transcript coding for the key enzymes involved in glutaminolysis, *GLSiso1*, *GLSiso2*, *GLS2*, *GLUD1*, *GOT1*, *GOT2*, and *GPT2* was performed in our series of astrocytomas of different malignant grades and NN brain samples. Interestingly, although no significant differential expression of the total *GLS* transcripts was observed among different grades of astrocytoma compared to NN, a significant *GLSiso2* (*GAC*) hyperexpression was observed in all grades of astrocytoma when compared to NN (*p* < 0.0001 Kruskal–Wallis test, and *p* < 0.001 Dunn test), with the highest expression levels detected in a set of GBM samples (Fig. 1a). Of note, *GLSiso2* expression increased in parallel to the grade of malignancy (*p* < 0.0001 AGII vs. AGIII, *p* < 0.05 AGII vs. GBM, *p* < 0.05 AGIII vs. GBM; Dunn test) which reflected an increase of its correlation with the gene expression levels of the glutaminolysis pathway from NN to GBM (Fig. 1a). When gene expression level correlations were analyzed, *GLSiso2* expression correlated weakly only with *GPT2* expression in NN, while no correlation was detected in AGI. *GLSiso2* correlated negatively with *GLS2*, *GOT1*, and *GOT2* in AGII and positively with *GLS2* and *GLSiso1* in AGIII, whereas *GLSiso2* correlated positively with all genes of the glutaminolysis pathway in GBM (Fig. 1b). All statistically significant values are demonstrated in Supplemental Table 1. Additionally, as shown in Fig. 2, the *GLSiso2* expression levels presented high discriminatory power to distinguish between GBM and NN samples by ROC curve analysis (AUC = 0.919; 95% CI, 0.867–0.971) and between GBM and AGII, although with lower discriminatory power (AUC = 0.675; 95% CI, 0.569–0.781).

In contrast, *GLSiso1 mRNA* expression was significantly lower in AGI, AGII, and GBM compared to NN (*p* < 0.0001 Kruskal–Wallis test, and *p* < 0.05 Dunn´s test) (Fig. 1a), with discriminatory power to distinguish between GBM and NN (AUC = 0.796; 95% CI, 0.1–0.308). No significant difference in its expression was detected in a pairwise comparison among different grades of astrocytoma. However, we observed a strong positive correlation between *GLSiso1* and *GLS2* in AGII and with *GLSiso2* in AGIII, as well as with *GLSiso2*, *GLUD1, GOT1*, *GOT2,* and *GPT2* in GBM cases. Similarly, *GLS2* hypoexpression was observed in astrocytoma of all malignant grades compared to NN (*p* < 0.0001 Kruskal-Wallis test, and *p* < 0.00001 for all astrocytoma grades and NN Dunn test), and its expression level presented the power to distinguish between GBM and NN (AUC = 0.791; 95% CI, 0.000–0.06) and to distinguish between GBM and AGII, but with lower discriminatory power (AUC = 0.65; 95% CI, 0.221–0.48).

**Fig. 3:**
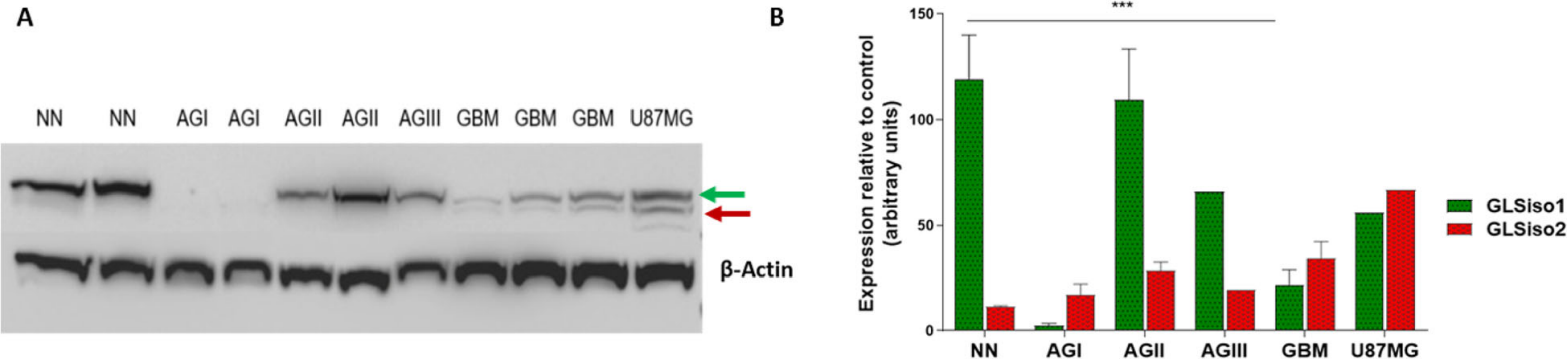
GLS isoforms expression profile during astrocytoma progression and U87MG human GBM cells. **a** Western blot analysis of the expression of GLS isoforms in non-neoplastic (NN), pilocytic astrocytoma (AGI), low-grade astrocytoma (AGII), anaplastic astrocytoma (AGIII), glioblastoma (GBM), and U87MG GBM cell line. GLSiso1 and GLSiso2 are indicated by green arrow and red arrows, respectively. β-actin was used as a loading control. **b** Quantification of the protein relative to β-actin protein by ImageJ, represented by mean values ± standard deviation. The graph is representative of at least two replicates of one experiment. ***p < 0.001, Two-way ANOVA, Bonferroni post-test

GLS isoforms expression was also investigated at the protein level, and we confirmed a differential expression of GLSiso1 and GLSiso2 in NN and astrocytomas of different malignant grades (Fig. 3). Whereas GLSiso1 was present in all NN and diffusely infiltrative astrocytoma (grade II to IV) samples, with higher abundance in NN samples in comparison to GBM samples (*p* < 0.001 ANOVA two–way, with Bonferroni post-test). In contrast, GLSiso2 was mostly detected in GBM cases and only slightly detected in NN samples. GLS isoforms were detected in the U87MG cell line—a GBM mesenchymal subtype cell line (Fig. 3).

### *GPT2* downregulation in the GBM mesenchymal subtype correlated to upregulation of genes involved in GSH synthesis

Once Glu is synthesized it can be converted to α–KG, an intermediate of the TCA cycle, by glutamate dehydrogenase (GLUD1) and glutamate transaminases, as glutamate oxaloacetate transaminases (GOT1–cytosolic and GOT2–mitochondrial) and glutamate pyruvate transaminases (GPT1–cytosolic and GPT2–mitochondrial), which transfer amino groups from oxaloacetate or from pyruvate to generate α–KG and aspartate or alanine, respectively [17]. The expression levels of *GLUD1*, *GOT1*, *GOT2*, and *GPT2* were differentially expressed in astrocytomas compared to NN (*p* < 0.0001 Kruskal–Wallis test for *GLUD1*, *GOT1*, and *GPT2* and *p* < 0.002 for *GOT2*) (Fig. 1a). Interestingly, *GLUD1* expression was significantly decreased in GBM compared to AGII (*p* < 0.005 Dunn test), and its expression level presented discriminatory power to distinguish between GBM and NN (ROC AUC = 0.770; 95% CI, 0.128–0.332) and between GBM and AGII (ROC AUC = 0.742; 95% CI, 0.147–0.368) (Fig. 2).

In contrast, *GOT2* expressions increased according to malignancy (*p* < 0.001 AGII vs. AGIII and *p* < 0.01 AGII vs. GBM, Dunn test) (Fig. 1a) and presented discriminatory power to distinguish between GBM and AGII (AUC = 0.709; 95% CI, 0.608–0.810) (Fig. 2). *GPT2* expression also increased significantly according to malignancy (AGI relative to AGII *p* < 0.001, to AGIII *p* < 0.0001, to GBM *p* < 0.05; AGII vs AGIII *p* < 0.05, AGIII vs GBM *p* < 0.005; Dunn test), besides, the only different expression level in comparison with NN was AGIII (*p* < 0.0001) (Fig. 1a). Although *GOT1* expression levels differed significantly between NN and astrocytoma, with discriminatory power to distinguish GBM from NN (ROC AUC = 0.902; 95% CI, 0.038–0.159), no significant difference of *GOT1* expression levels was detected among the astrocytoma grades of malignancy (Fig. 1a).

Considering the natural history of malignancy progression of malignancy from AGII to GBM, an upregulation of *GLSiso2*, *GOT2*, and *GPT2* expression levels were observed, in contrast to the downregulation of *GLUD1*. However, a large spreading of their expressions was detected in GBM, consistent with the well-known heterogeneity observed in GBM. Therefore, we analyzed the expression levels in GBM cases classified according to the molecular subtypes in proneural (PN), classical (CS), and mesenchymal (MS) subtypes [40]. Our cohort comprised 14 PN, 38 CS, and 14 MS cases. We found a statistical difference for *GLS2* expression among the groups (*p* < 0.005, Kruskal–Wallis test) and comparing two groups: PN vs MS (*p* < 0.05, Dunn test) and CS vs MS (*p* < 0.05, Dunn test), with lower expression detected in the MS subtype (Supplemental Figure 1).

**Fig. 4:**
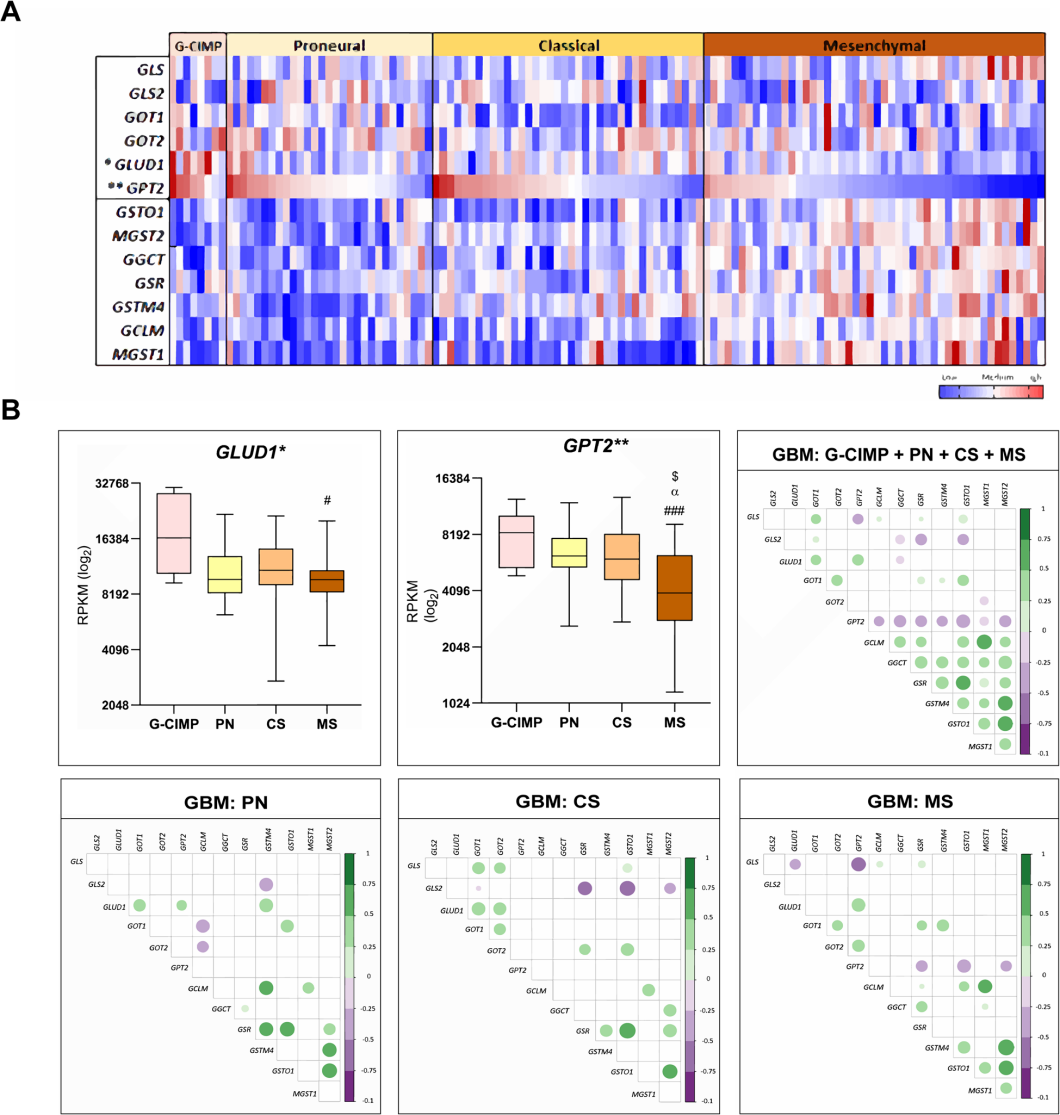
TCGA dataset: glutaminolysis- and GSH synthesis-related gene expressions and their correlation in GBM subtypes. **a** Heatmap representing gene expression levels in G-CIMP, proneural (PN), classical (CS), mesenchymal (MS) GBM subtypes. Upregulated values are in red and downregulated in blue. The RPKM values were normalized by *z*-score (*p = 0.05, **p < 0.0001, Kruskal-Wallis test). **b** Box and whiskers plot of *GLUD1* and *GPT2* expression levels in different GBM subgroups. The lines in the middle of the boxes show the median expression in each group, and the top and the bottom of the boxes represent the first and third quartiles. The results are presented in the log_2_ scale of RPKM values. (Kruskal-Wallis: *p < 0.05, **p < 0.005, Dunn test: MS vs PN (α); MS vs CS ($); G-CIMP vs MS: (#). Spearman correlation matrices of gene expression levels in different GBM molecular subtypes. The color bar on the right indicates the level of correlation (*r*) ranging from dark orange (negative correlation) to purple (positive correlation). The color intensiveness and the circle size are proportional to *r* values. Only the correlations with *p* < 0.05 are plotted

To validate these findings, we analyzed gene expression in silico in a larger database. The TCGA GBM database with gene expression from RNAseq comprising 37 PN (8 G-CIMP and 29 non-G-CIMP), 38 CS, and 48 MS cases. *GPT2* expression levels varied significantly among the GBM subtypes (*p* < 0.0001, Kruskal–Wallis test) with lower levels in MS than G-CIMP (*p* < 0.0005), PN (*p* < 0.05), and CS (*p* < 0.05, Dunn test). *GLUD1* expression levels also varied significantly amongst GBM subtypes (*p* = 0.05, Kruskal–Wallis test) with significant higher levels in G-CIMP compared to PN (*p* < 0.01) and to MS (*p* < 0.005, Dunn test). Although no statistical significance was observed of *GLS* expression among GBM subtypes, a trend of increase of its expression was noted in the MS subtype (Fig. 4a). The expression levels of *GLS* isoforms were not available in this dataset. The TCGA data analysis showed the downregulation of *GLUD1* and *GPT2* involved in the conversion of Glu to α–KG. *GLUD1* differed statistically in G–CIMP to MS (*p* < 0.01). Particularly, *GPT2* differed when comparing all groups with the MS subtype of GBM (G–CIMP–MS *p* < 0.001; PN–MS *p* < 0.01; CS–MS *p* < 0.05).

The downregulation of both genes *GLUD1* and *GPT2* suggest that the intracellular availability of Glu is increased, especially in the MS subtype of GBM, which led us to investigate another important Glu metabolism pathway: GHS. To this purpose, we selected the genes related to GSH pathway, glutamate-cysteine ligase modifier subunit (*CGLM*), gamma–glutamylcyclotransferase (*GGCT*), glutathione S–transferase mu 4 (*GSTM4*), glutathione S–transferase omega 1 (*GSTO1*), microsomal glutathione S–transferase 1 (*MGST1*), and microsomal glutathione S–transferase 2 (*MSGT2*) and analyzed the expression levels in the GBM database of TCGA (Fig. 4a). Additionally, the expression values were correlated to the expression data of glutaminolysis genes (Fig. 4b). The seven genes related to GSH synthesis presented differential expression levels among the GBM molecular subtypes, with statistical differences for all genes (*p* < 0.0005, Kruskal–Wallis test). Particularly, the expression of these genes was higher in the MS subtype compared to the other subtypes (Supplemental Figure 2). Moreover, the gene expression levels of GSH synthesis were highly correlated among themselves when all groups of GBM were analyzed together (GBM: G–CIM+PN+CS+MS), and interestingly, an inverse correlation was noted with *GPT2* expression. Particularly in the MS subtype, *GPT2* expression correlated inversely to the expression levels of *GSTO1, GSR,* and *MGST2*, suggesting the possibility of Glu not converted to α–KG being used for GHS synthesis (Fig. 4b). All statistically significant values are demonstrated on Supplemental Table 2.

### *GLUD1* upregulation in *IDH1*^mut^ AGIII correlated to downregulation of genes involved in GSH synthesis

**Fig. 5:**
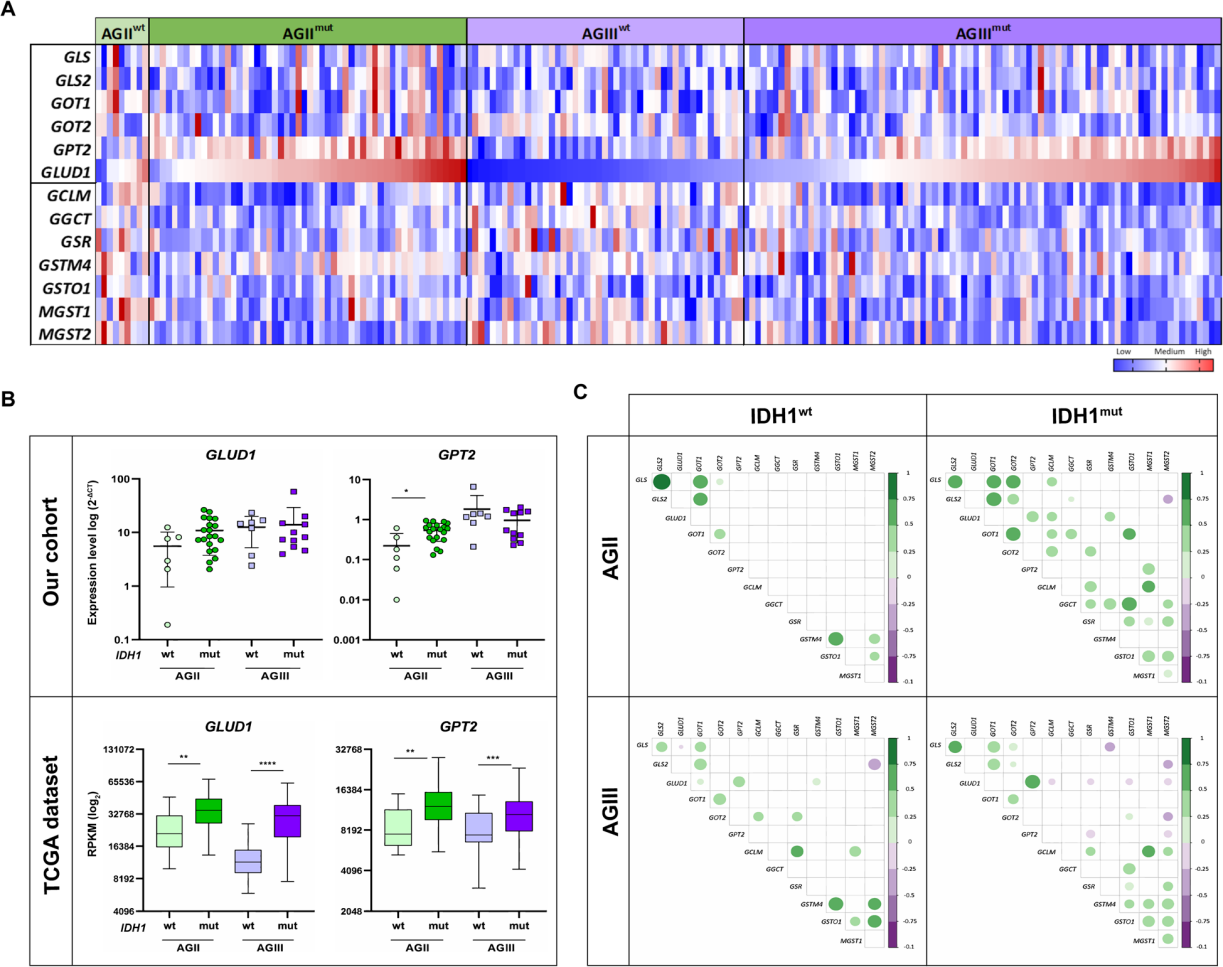
AGII, AGIII TCGA dataset: glutaminolysis- and GSH synthesis-related gene expressions according to *IDH1* mutation. **a** Heatmap representing the expression levels of genes presenting statistical significance in AGII and AGIII with wild type (wt) and mutated *IDH1* (mut). Upregulated values are represented in red and downregulated in blue. The RPKM values were normalized by *z*-score. **b** The differential expression levels of *GLUD1* and *GPT2* in each stratified group. (Mann-Whitney test: *p < 0.05, **p < 0.01; ***p < 0.0005, ****p < 0.0001). Horizontal bars show the median expression in each group for the up panels, while the bottom panel boxes represent the first (top) and third (bottom) quartiles, and the median is represented by the middle line in the boxes. The results are presented in the log_2_ scale of RPKM values. **c** Spearman correlation matrix among the gene expression levels of each group. The color bar on the right indicates the level of correlation ranging from dark orange (negative correlation) to blue (positive correlation). The color intensiveness and the circle sizes are proportional to r values. Only the correlations with *p* < 0.05 are plotted

G-CIMP cases of PN molecular subtype of GBM presented the higher *GLUD1* and *GPT2* expression levels when compared to the other subgroups. Additionally, genes related to GSH synthesis presented the lowest expression levels in G-CIMP cases. These data and the information that increased conversion of Gln to Glu has been described in glioma cells harboring *IDH1* mutation [41] motivated us to investigate the *IDH*1 mutation status influence in the expression levels of genes involved in the glutaminolysis pathway and GSH. Gene expression levels previously analyzed in GBM cases were also analyzed in AGII and AGIII cases of TCGA, separating cases with and without *IDH1* mutation (Fig. 5a). In our cohort, 20 AGII out of 26 cases (77%) presented *IDH1* mutation, and 11 out of 18 AGIII cases harbored *IDH1* mutation (61%). Interestingly, upregulated *GLUD1* and *GPT2* expressions were observed in *IDH1*^mut^ AGII cases, with a significant difference compared to *IDH1*^wt^ AGII cases for *GPT2* expression (*p* < 0.05, Mann-Whitney test), and a trend of increase for *GLUD1* (Fig. 5b). In a larger TCGA dataset, with 51 *IDH1*^mut^ AGII out of 63 cases (86%), and 80 *IDH1*^mut^ AGIII out of 129 cases (63%), a significant higher *GLUD1* and *GPT2* expression levels were observed both in AGII and AGIII harboring *IDH1* mutation when compared to cases without *IDH1* mutation (*p* < 0.01 and *p* < 0.0001 for *GLUD1* in AGII and AGIII respectively; *p <* 0.01 and *p* < 0.001 for *GPT2* in AGII and AGIII, respectively, Mann-Whitney test) (Fig. 5b). Expression analyses of expression levels of all genes presented in the correlation matrix demonstrated that *IDH1*^mut^ AGII cases presented activation of both glutaminolysis and GSH synthesis in contrast to *IDH1*^wt^ AGII, with an inverse correlation between *GLS2* and *MSGT2* expressions in *IDH1*^mut^ AGII (Fig. 5c). On the other hand, *IDH1*^mut^ AGIII cases presented a significantly high correlation between *GLUD1* and *GPT2* expression levels, and inverse correlations with several genes related to GSH synthesis. Of note, the *GLUD1* expression level was inversely correlated to *GSR*, *GCLM, GSTO1*, and *GSMT2* expression levels, indicating the downregulation of these gene expressions when *GLUD1* was upregulated in AGIII cases with *IDH1* mutation (Fig. 5c). All statistically significant values are demonstrated on Supplemental Table 3.

### GLUD1 and GPT2 protein downregulation in GBM-MS and GLUD1 downregulation in AGII-IDH^wt^ correlated with upregulation of GS activity

**Fig. 6:**
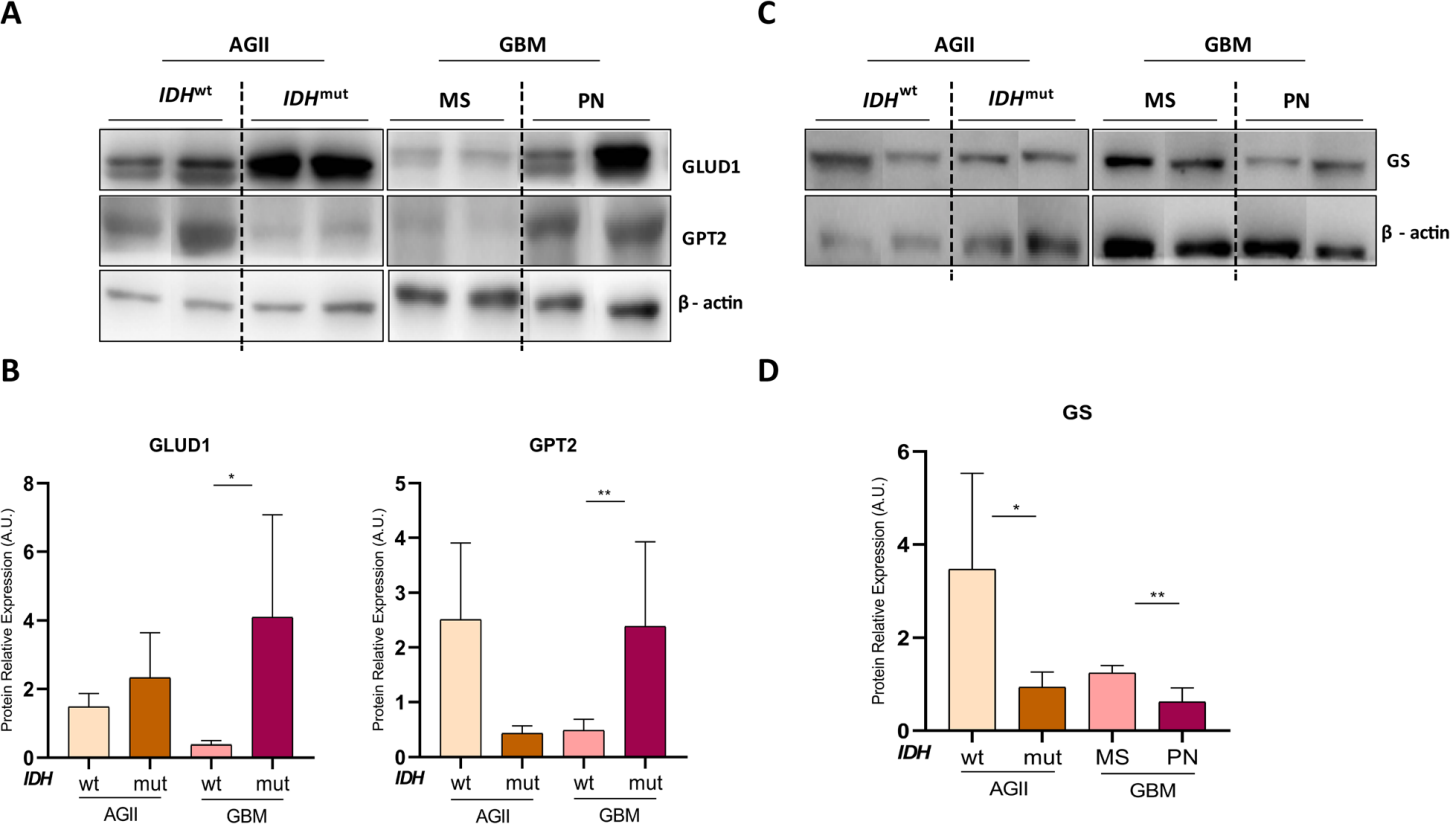
GLUD1, GPT2, and GS protein expression analysis in GBM and low-grade astrocytomas according to IDH mutation status. **a** Western blotting analysis of the expression of GLUD1 and GPT2 in AGII-*IDH*^wt^, AGII-*IDH*^mut^, GBM-MS (*IDH*^wt^) and GBM-PN (*IDH*^mut^) samples, and GS in **c**. β-actin was used as the loading control. **b**, **d** Quantification of each protein relative to β-actin protein by ImageJ, represented by mean values ± standard deviation. The graph is representative of at least four replicates of one experiment. *p < 0.05, **p < 0.001, one-way ANOVA, Bonferroni post-test

The GBM-MS presented low expression of GLUD1 and GPT2 protein levels when compared to the GBM-PN cases (*p* < 0.05 and *p <* 0.001, respectively). Although statistical significance was not reached, AGII-IDH^wt^ presented low GLUD1 protein level (Fig. 6a, b). Additionally, we evaluated whether the level of these proteins correlated with glutathione synthetase (GS) expression. Interestingly, a significant increase of GS expression was observed in GBM-MS and AGII-*IDH*^wt^ in comparison with GBM-PN and AGII-*IDH*^mut^ (*p* < 0.01 and *p* = 0.05, respectively) (Fig. 6c, d). We also found a trend of increased levels of GSH in GBM-MS (*IDH*^wt^) samples in contrast to more uniform low GSH levels in GBM-PN (*IDH*^mut^) (Supplemental Figure 3).

## Discussion

Metabolic reprogramming has been proposed to be a hallmark of cancer [42], and in the present analysis, we observed a progressive activation of the glutaminolysis from low-grade astrocytoma to GBM. Glutaminolysis has been pointed to as one of the major altered metabolic pathways related to tumor growth [12, 16]. High extracellular Gln concentration has been associated with cell transformation [17], and its metabolism was related to cell survival and tumor growth by maintaining redox balance, bioenergetics, and supporting macromolecular biosynthesis [28, 42]. We have previously reported the upregulation of Gln transporters, *ASCT2* and *LAT1*, in all grades of astrocytoma [20]. Here, we showed the upregulation of the mitochondrial isoform of *GLS* [39], *GLSiso2* (*GAC*), in all grades of astrocytoma at gene and protein levels, and a gradual increase of its expression was observed in parallel to the increment of malignancy. GLSiso2 involvement in cancer progression has been previously reported in prostate cancer and B cell lymphoma [43]. GLSiso2 is activated by inorganic phosphate [39] and it is also under c–*Myc* oncogene influence, through a mechanism involving miRNA [43, 44]. The c-Myc can also upregulate the GLS isoforms KGA and GAC at protein levels increasing the levels of intracellular glutamate in Epstein-Barr virus-infected cells [68]. Oscillation of *GLSiso2* expression has been associated with oxygen concentration, with an increase in hypoxic conditions [39]. Our finding of *GLSiso2* higher expression in GBM, the more malignant astrocytoma presenting necrosis, corroborated these previous observations. The cytosolic *GLSiso1* (*KGA*) expression was also higher in more malignant than lower-grade astrocytomas. Nevertheless, the high expression observed in NN tissue renders this target less eligible for therapeutic purposes.

The other glutaminase, *GLS2,* in contrast to *GLS* with broad distribution among normal tissue, presents a more restricted distribution in the liver, brain, pituitary gland, and pancreas [45, 46]. The *GLS2* expression level was significantly lower in astrocytoma than NN, with the lowest expression in GBM of MS subtype in our cohort. This finding corroborates the tumor suppressor role attributed to *GLS2* in previous studies, where inhibition of tumor cell proliferation, colony formation, and migration were attributed to *GLS2* [28, 43, 47–51]. This tumor suppressor activity is dependent on p53 and other related proteins, as p63 and p73 [48]. Therefore, concerning the first step of the glutaminolysis pathway, our findings suggested that *GLSiso2* plays a key role in tumorigenesis and malignant progression of astrocytoma, whereas the *GLS2* expression pattern is consistent with tumor suppressor function, being mostly suppressed in the aggressive MS molecular subtype of GBM.

Interestingly, the downflow activation of the glutaminolysis pathway with the conversion of Glu to α–KG through dehydrogenase and transaminase varied according to the astrocytoma grade. Significant downregulation of *GLUD1* and *GPT2* expressions were observed in GBM compared to lower-grade astrocytoma in our cohort and confirmed in the TCGA dataset. Particularly, *GPT2* was significantly downregulated in GBM of MS subtype compared to other molecular subtypes. We also observed this downregulation of GLUD1 and GPT2 at the protein level in GBM-MS subtype. The downregulation of these proteins may increase intracellular Glu availability, which may be directed for GSH synthesis [52].

GSH is a tripeptide formed by glutamic acid, cysteine, and glycine and plays an important role in the maintenance of the intracellular redox balance [53, 54]. Elevated GSH levels confer resistance to chemotherapy in various types of cancer [55–57] by binding to or reacting with drugs, interacting with ROS, preventing damage to proteins or DNA, and participating in DNA repair processes [55]. Moreover, GSH- and GSH-related enzymes including synthetase (GS), ligase (GCLM), transferase (GGT), reductase (GSR), and glutathione S–transferases (GSTM4, GSTO1, MGST1, MGST2) activities may play a role in adaptive detoxification processes in response to the oxidative stress, thus contributing to drug resistance phenotype [53, 54].

The increase of intracellular Glu level may favor its release to the extracellular space by a Gln/cysteine antiporter system x c–dependent, which increases intracellular cysteine levels ([Cys]i). In turn, high [Cys]i favors GSH synthesis [52]. The TCGA data analysis showed a significant inverse correlation among *GPT2* expression and expression level of several genes related to GSH synthesis. Particularly, upregulation of *GSTO1*, *MGST2,* and *GSR* were correlated significantly to the downregulation of *GPT2* in the MS subtype of GBM. Also, we observed a significant increase of the GS protein and a trend of an increase of GSH protein level in the GBM-MS in comparison with the GBM-PN samples. The antioxidative effect provided by increased synthesis of GSH can balance the elevated generation of ROS due to the high metabolic rate presented by GBM cells and favor their survival [58]. Such mechanism may be related to the aggressive behavior of GBM of MS molecular subtype.

In contrast, AGII and AGIII presented higher *GLUD1* expression levels than GBM*,* and particularly in those cases harboring *IDH1* mutation, and a similar trend was observed at the protein level, although this observation needs to be extended for additional cases to reach statistical significance. Similarly, *GPT2* expression was also higher in AGII and AGIII cases harboring *IDH1* mutation, but this finding was not confirmed at the protein level in our studied cohort. However, GS protein level was significantly lower in AGII-IDH^mut^ compared to AGII-IDH^wt^, suggesting that low-grade astrocytomas harboring IDH mutation may be more susceptible to ROS induced stress. Metabolomic studies of *IDH1* mutant cells have revealed alterations in Gln, fatty acid, and citrate synthesis pathways [59, 60]. *IDH1* mutation was shown to convert α–KG to D–2–hydroxyglutarate, which due to its structural similarities acts as a competitive inhibitor reducing the activity of α–KG–processing enzymes [61]. As feedback, α–KG is replenished by glutaminolysis and TCA cycle, which leads to a decrease in Gln and Glu levels [62]. Therefore, *IDH*^mut^ gliomas are “glutamate addicted”, and the lack of Glu decreases its exchange with Cys through the system X_C_^–^ [63]. The lack of cytoplasmic Cys reduces GSH synthesis, which increases the susceptibility to ROS–induced stress as through radiation therapy or TMZ treatment [63]. In this context, reduced Glu contributes to a better outcome presented by gliomas with *IDH1* mutation [41, 52]. Our correlation analysis among genes related to glutaminolysis and GSH synthesis-related genes demonstrated that *GLUD1* and *GPT2* expression levels inversely correlated to GSH synthesis-related gene expression levels, particularly in *IDH1*^mut^AGIII. Our findings reinforce the hypothesis that decreasing Glu may sensitize *IDH1*^mut^ cells to radiation and ROS-inducing drugs due to reduced GSH synthesis. Indeed, GLS inhibition and *IDH1* mutation were recently demonstrated to present a synthetic lethal relationship under conditions of oxidative stress [41].

Our findings together with TCGA data analysis indicated that AGII and AGIII harboring *IDH1* mutation may decrease tumor cell fitness by lowering Glu, GSH, and resistance to oxidative stress. Interestingly, the end metabolites of these enzymes, ammonia, and alanine are measurable by the MR spectroscopy [64–67]. Thus, monitoring the waning of *GLUD1* and *GPT2* expression levels by measuring their end substrates by this non-invasive imaging technique may potentially detect the progression of lower-grade astrocytomas harboring *IDH1* mutation towards secondary GBM, and it would, therefore, allow a change in the therapeutic strategy for these patients. Such hypothesis would be worthwhile to test in future studies.

## Conclusion

**Fig. 7:**
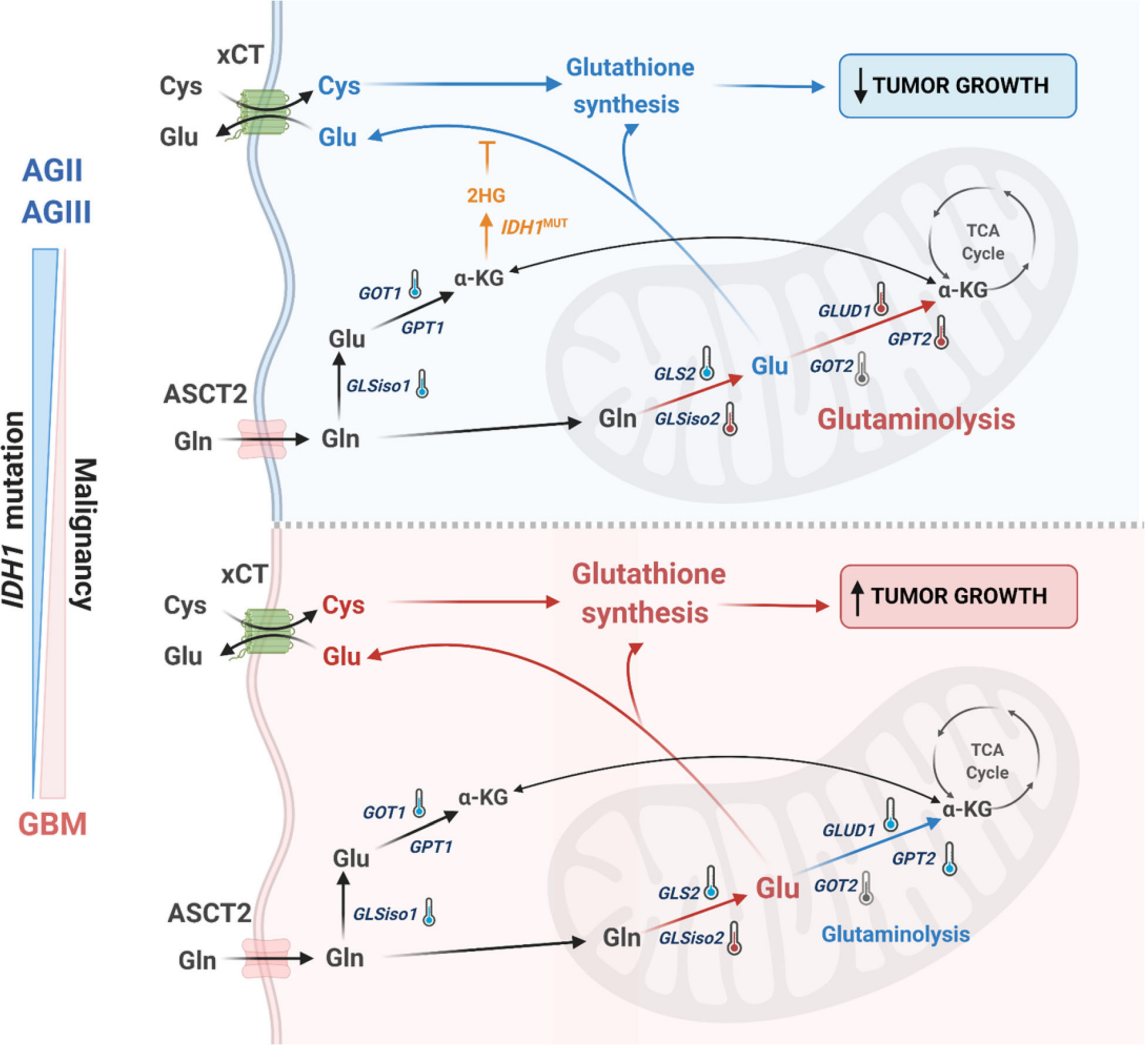
Schematic representation of glutaminolysis genes differentially expressed in GBM. GBM cases presented hyperexpression of *GLSiso2* and hypoexpression of other genes of the glutaminolysis pathway (*GLSiso1*, *GLS2*, *GLUD1*, *GPT2*), (downregulation of GLUD1 and GPT2 was confirmed at protein level) leading to an accumulation of Glu and activation of GSH synthesis and consequently inducing tumor cell proliferation and survival. On the other hand, AGII and AGIII *IDH1*^mut^ display hyperexpression of *GLSiso2*, *GLUD1,* and *GPT2* (upregulation of GLUD1 was confirmed at protein level) leading to activation of glutaminolysis pathway and fueling the TCA cell cycle. Additionally, in *IDH1*^mut^ cases, a decrease in Glu availability sensitizes the tumor cells to oxidative stress leading to slow tumor growth. The downregulated expression is represented by blue thermometers, upregulated by red thermometers, and the non-significant differential expression in gray. Red arrows represent the activation of the pathway and blue arrows represent the inactivation of the pathway

In conclusion, *GLSiso2* upregulation was associated with tumorigenesis and tumor progression in astrocytomas. Particularly in GBM, the accumulation of Glu due to *GPT2* and *GLUD1* downregulation correlated to upregulation of genes related to GSH synthesis which could favor tumor cell survival, mostly in the most aggressive MS subtype. In contrast, *GLUD1* may lead to a decrease in GSH synthesis in *IDH1*^mut^ low-grade astrocytomas increasing the susceptibility to oxidative stress, rendering them more sensitive to radiation therapy and to alkylating therapy (Fig. 7).

## Supplementary Information

**Additional file 1: Figure S1**. Expression analysis of genes related to glutaminolysis in different molecular subtypes of GBM in our cohort. A: Heatmap of *GLSiso1*, *GLSiso2*, *GLS*, *GLS2*, *GOT1, GOT2, GPT2*, and *GLUD1* mRNA expression levels in different molecular subtypes of GBM (PN: proneural, CS: classical, MS: mesenchymal). Upregulated values are in red and downregulated in blue. The RPKM values were normalized by z-score. B: *GLS2* expression differed significantly among the subtypes (Kruskal-Wallis, *p* < 0.005, **p < 0.05, Dunn test). Horizontal bars show the median relative expression in each group.

**Additional file 2: Figure S2**. Expression analysis of GPT2 and genes related to glutathione synthesis in different molecular subgroups of GBM from TCGA RNAseq dataset. Box and whiskers plot of *GPT2, GCLM, GGCT, GSR, GSTM4, GSTO1, MGST1*, and *MGST2* expression levels in G-CIMP, proneural (PN), classical (CS), and mesenchymal (MS) GBM cases. The top and the bottom of boxes represent the first and third quartiles, respectively, and the lines in the middle the median of the groups. Kruskal-Wallis: *p < 0.05, **p < 0.005, ***p < 0.0005, Dunn test: CS vs PN (#); MS vs PN (¶); MS vs CS ($); G-CIMP vs MS: (‡); G-CIMP vs CS: (@). The results are presented in the log2 scale of RPKM values.

**Additional file 3: Figure S3** Analysis of Reduced Glutathione (GSH) levels in AGII*IDH*^wt^_,_ AGII*IDH*^mut^, GBM-MS (*IDH*^wt^) and PN (*IDH*^mut^). GSH levels [pmol/μg of total protein] analysis of low-grade astrocytoma IDH^wt^, low grade astrocytoma *IDH*^mut^, Glioblastoma^wt^ –Mesenchymal, and glioblastoma IDH^mut^ –Proneural). Results are presented as mean ± standard deviation (n ≥ 3 samples of each group).

**Additional file 4: Supplemental Table S1**. Correlations values of the glutaminolysis pathway genes in all grades of astrocytoma.

**Additional file 5: Supplemental Table S2**. Correlations values of the glutaminolysis and GSH pathway genes in the subtypes of GBM.

**Additional file 6: Supplemental Table S3**. Correlations values of the glutaminolysis and GSH pathway genes in AGII and AGIII with *IDH1* wild type (*IDH1*^wt^) and mutated *IDH1* (*IDH1*^mut^).

## Data Availability

Normalized gene expression data are available from the corresponding author on request. The results shown here are in part based upon data generated by the TCGA Research Network: https://www.cancer.gov/tcga.

## Abbreviations

GBM: Glioblastoma
AGI: Astrocytoma grade I
AGII: Astrocytoma grade II
AGIII: Astrocytoma grade III
NN: Non-neoplastic
Gln: Glutamine
Glu: Glutamate
TCA: Tricarboxylic acid
α–KG: Alpha ketoglutarate
GSH: Glutathione
GLS: Glutaminase
GLS2: Glutaminase 2
GLSiso1: Glutaminase isoform 1
GLSiso2: Glutaminase isoform 2
GLUD1: Glutamate dehydrogenase 1
GOT1: Glutamate oxaloacetate transaminase 1
GOT2: Glutamate oxaloacetate transaminase 2
GPT1: Glutamate pyruvate transaminase 1
GPT2: Glutamate pyruvate transaminase 2
TCGA: The Cancer Genome Atlas
IDH: Isocitrate dehydrogenase
IDH1: Isocitrate dehydrogenase 1
IDH1^MUT^: Isocitrate dehydrogenase 1 mutation
IDH^WT^: Isocitrate dehydrogenase wild type
CNS: Central nervous system
WHO: World Health Organization
NF1: Neurofibromin 1
RB1: Retinoblastoma 1
TMZ: Temozolomide
qRT–PCR: Quantitative reverse transcription PCR
ASCT2 (SLC1A5): Solute carrier family 1 member 5
LAT1 (SLC7A5): Solute carrier family 7 member 5
HPRT: Hypoxanthine phosphoribosyltransferase
GUSβ: Glucuronidase beta
TBP: TATA box binding protein
PCR: Polymerase chain reaction
ATCC: American Type Culture Collection
mRNA: Messenger RNA
ROC: Receiver operating characteristic
PN: Proneural
CS: Classical
MS: Mesenchymal
G–CIMP: Glioma CpG island methylator phenotype
CGLM: Glutamate–cysteine ligase modifier subunit
GGCT: Gamma–glutamylcyclotransferase
GSTM4: Glutathione S–transferase mu 4
GSTO1: Glutathione S–transferase omega 1
MGST1: Microsomal glutathione S–transferase 1
MGST2: Microsomal glutathione S–transferase 2
GSR: Glutathione–disulfide reductase
GS: Glutathione synthetase
ROS: Reactive oxygen species
MR: Magnetic resonance
