# Supplementary figures and images for: Glutaminolysis dynamics during astrocytoma progression correlates with tumor aggressiveness
Source: Cancer Metab. 2021 Apr 28;9:18. doi: 10.1186/s40170-021-00255-8 (PMC8082835; doi:10.1186/s40170-021-00255-8)

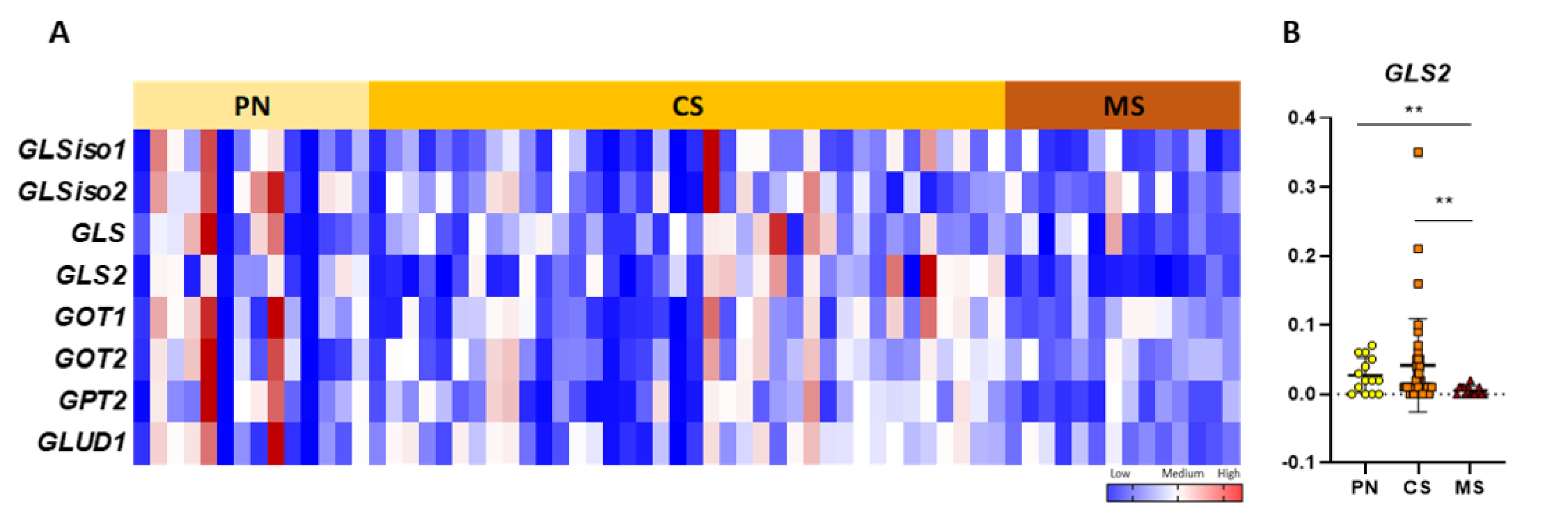

Supplement: Supplementary file 1 — Additional file 1: Figure S1. Expression analysis of genes related to glutaminolysis in different molecular subtypes of GBM in our cohort. A: Heatmap of GLSiso1, GLSiso2, GLS, GLS2, GOT1, GOT2, GPT2, and GLUD1 mRNA expression levels in different molecular subtypes of GBM (PN: proneural, CS: classical, MS: mesenchymal). Upregulated values are in red and downregulated in blue. The RPKM values were normalized by z-score. B: GLS2 expression differed significantly among the subtypes (Kruskal-Wallis, p < 0.005, **p < 0.05, Dunn test). Horizontal bars show the median relative expression in each group. [file 40170_2021_255_MOESM1_ESM.tif]

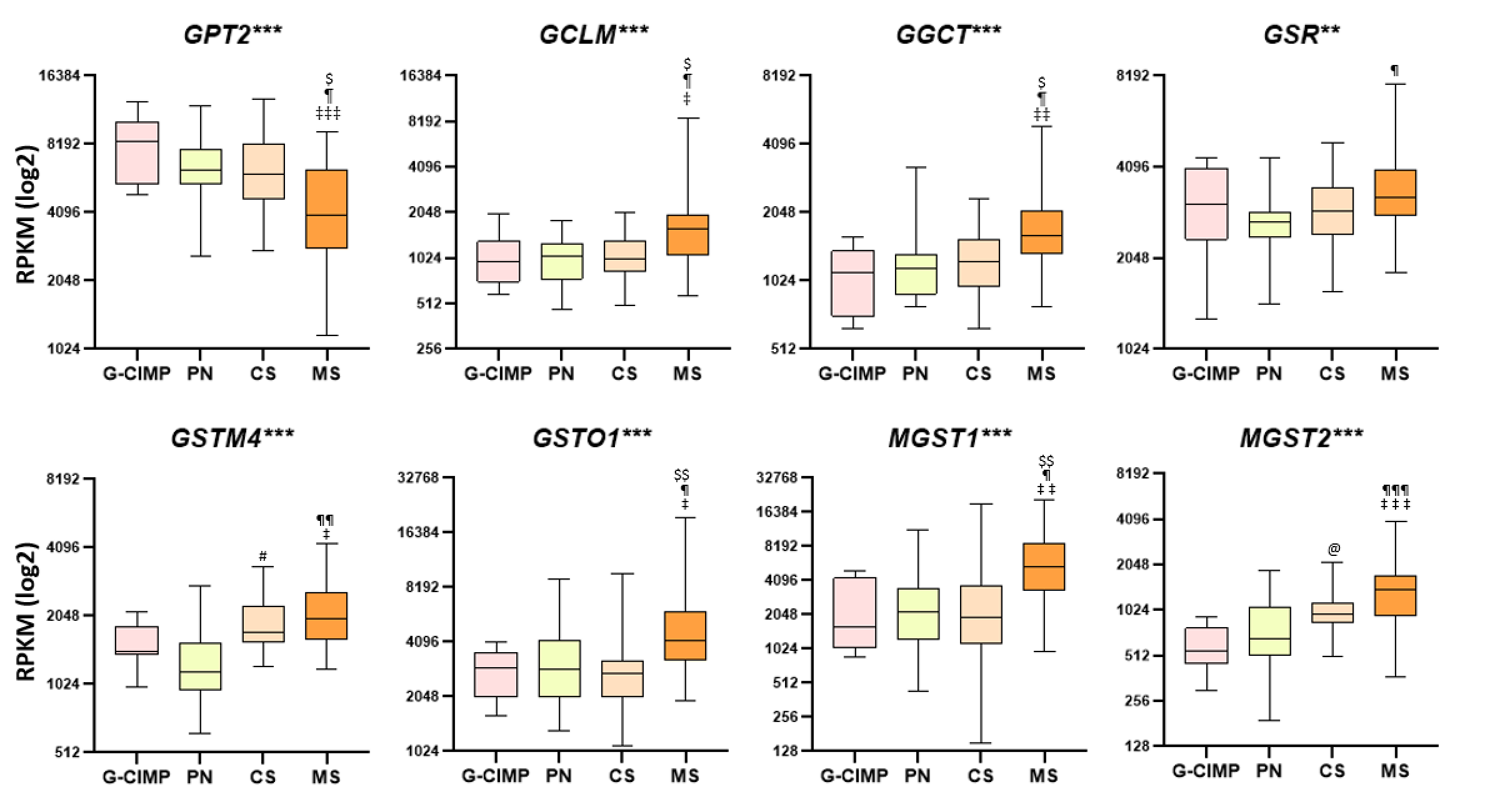

Supplement: Supplementary file 2 — Additional file 2: Figure S2. Expression analysis of GPT2 and genes related to glutathione synthesis in different molecular subgroups of GBM from TCGA RNAseq dataset. Box and whiskers plot of GPT2, GCLM, GGCT, GSR, GSTM4, GSTO1, MGST1, and MGST2 expression levels in G-CIMP, proneural (PN), classical (CS), and mesenchymal (MS) GBM cases. The top and the bottom of boxes represent the first and third quartiles, respectively, and the lines in the middle the median of the groups. Kruskal-Wallis: *p < 0.05, **p < 0.005, ***p < 0.0005, Dunn test: CS vs PN (#); MS vs PN (¶); MS vs CS ($); G-CIMP vs MS: (‡); G-CIMP vs CS: (@). The results are presented in the log2 scale of RPKM values. [file 40170_2021_255_MOESM2_ESM.tif]

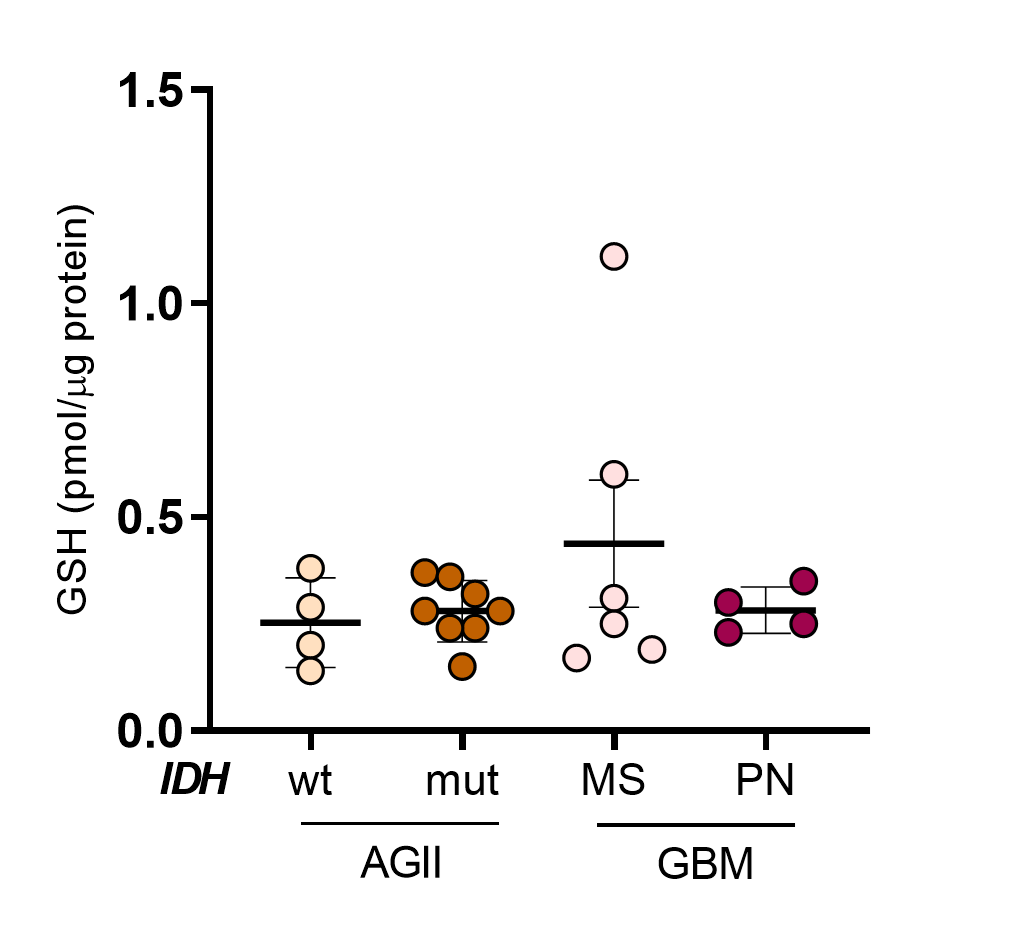

Supplement: Supplementary file 3 — Additional file 3: Figure S3 Analysis of Reduced Glutathione (GSH) levels in AGIIIDHwt, AGIIIDHmut, GBM-MS (IDHwt) and PN (IDHmut). GSH levels [pmol/μg of total protein] analysis of low-grade astrocytoma IDHwt, low grade astrocytoma IDHmut, Glioblastomawt –Mesenchymal, and glioblastoma IDHmut –Proneural). Results are presented as mean ± standard deviation (n ≥ 3 samples of each group). [file 40170_2021_255_MOESM3_ESM.tif]
