# Supplementary material for: Glutaminolysis dynamics during astrocytoma progression correlates with tumor aggressiveness
Source: Cancer Metab. 2021 Apr 28;9:18. doi: 10.1186/s40170-021-00255-8 (PMC8082835; doi:10.1186/s40170-021-00255-8)
Supplement: Supplementary file 4 — Additional file 4: Supplemental Table S1. Correlations values of the glutaminolysis pathway genes in all grades of astrocytoma. [file 40170_2021_255_MOESM4_ESM.docx]

**Supplemental Table 1. Correlations values of the glutaminolysis pathway genes in all grades of astrocytoma.**

|  |  |  |  |  |  |  |  |  |  |  |  |  |  |  |  |  |
| --- | --- | --- | --- | --- | --- | --- | --- | --- | --- | --- | --- | --- | --- | --- | --- | --- |
|  |  | **NN** | | | | | | | | | | | | | | |
|  | **GLSiso1** | | **GLSiso2** | |  | **GLS2** | | **GLUD1** | | **GOT1** | | **GOT2** | | **GPT2** | |  |
|  | **r** | **p** | **r** | **p** | **r** |  | **p** | **r** | **p** | **r** | **p** | **r** | **p** | **r** | **p** |  |
| **GLSiso1** | 1 |  |  |  |  |  |  |  |  |  |  |  |  |  |  |  |
| **GLSiso2** |  |  | 1 |  |  |  |  |  |  |  |  |  |  |  |  |  |
| **GLS2** | 0.72 | 0.02 |  |  | 1 |  |  |  |  |  |  |  |  |  |  |  |
| **GLUD1** |  |  |  |  | 0.63 |  | 0.00 | 1 |  |  |  |  |  |  |  |  |
| **GOT1** | 0.77 | 0.01 |  |  | 0.87 |  | 0.00 | 0.71 | 0.00 | 1 |  |  |  |  |  |  |
| **GOT2** | 0.76 | 0.01 |  |  | 0.81 |  | 0.00 | 0.7 | 0.02 | 0.93 | 0.00 | 1 |  |  |  |  |
| **GPT2** | 0.71 | 0.00 | 0.25 | 0.04 |  |  |  |  |  | 0.5 | 0.04 | 0.6 | 0.01 | 1 |  |  |
|  |  | **AGI** | | | | | | | | | | | | | | |
|  | **GLSiso1** | | **GLSiso2** | |  | **GLS2** | | **GLUD1** | | **GOT1** | | **GOT2** | | **GPT2** | |  |
|  | **r** | **p** | **r** | **p** | **r** |  | **p** | **r** | **p** | **r** | **p** | **r** | **p** | **r** | **p** |  |
| **GLSiso1** | 1 |  |  |  |  |  |  |  |  |  |  |  |  |  |  |  |
| **GLSiso2** |  |  | 1 |  |  |  |  |  |  |  |  |  |  |  |  |  |
| **GLS2** | 0.53 | 0.00 |  |  | 1 |  |  |  |  |  |  |  |  |  |  |  |
| **GLUD1** |  |  |  |  |  |  |  | 1 |  |  |  |  |  |  |  |  |
| **GOT1** | 0.48 | 0.00 |  |  | 0.48 |  | 0.02 | 0.63 | 0.01 | 1 |  |  |  |  |  |  |
| **GOT2** | 0.42 | 0.00 |  |  |  |  |  | 0.72 | 0.00 | 0.82 | 0.00 | 1 |  |  |  |  |
| **GPT2** |  |  |  |  |  |  |  | 0.83 | 0.00 | 0.68 | 0.00 | 0.77 | 0.00 | 1 |  |  |
|  |  | **AGII** | | | | | | | | | | | | | | |
|  | **GLSiso1** | | **GLSiso2** | |  | **GLS2** | | **GLUD1** | | **GOT1** | | **GOT2** | | **GPT2** | |  |
|  | **r** | **p** | **r** | **p** | **r** |  | **p** | **r** | **p** | **r** | **p** | **r** | **p** | **r** | **p** |  |
| **GLSiso1** | 1 |  |  |  |  |  |  |  |  |  |  |  |  |  |  |  |
| **GLSiso2** |  |  | 1 |  |  |  |  |  |  |  |  |  |  |  |  |  |
| **GLS2** | 0.73 | 0.01 | -0.58 | 0.00 | 1 |  |  |  |  |  |  |  |  |  |  |  |
| **GLUD1** |  |  |  |  |  |  |  | 1 |  |  |  |  |  |  |  |  |
| **GOT1** | 0.44 | 0.00 | -0.53 | 0.01 | 0.47 |  | 0.02 |  |  | 1 |  |  |  |  |  |  |
| **GOT2** |  |  | -0.37 | 0.04 |  |  |  | 0.63 | 0.01 | 0.69 | 0.00 | 1 |  |  |  |  |
| **GPT2** |  |  |  |  |  |  |  | 0.48 | 0.01 |  |  |  |  | 1 |  |  |
|  |  | **AGIII** | | | | | | | | | | | | | | |
|  | **GLSiso1** | | **GLSiso2** | |  | **GLS2** | | **GLUD1** | | **GOT1** | | **GOT2** | | **GPT2** | |  |
|  | **r** | **p** | **r** | **p** | **r** |  | **p** | **r** | **p** | **r** | **p** | **r** | **p** | **r** | **p** |  |
| **GLSiso1** | 1 |  |  |  |  |  |  |  |  |  |  |  |  |  |  |  |
| **GLSiso2** | 0.61 | 0.01 | 1 |  |  |  |  |  |  |  |  |  |  |  |  |  |
| **GLS2** | 0.51 | 0.00 | 0.3 | 0.04 | 1 |  |  |  |  |  |  |  |  |  |  |  |
| **GLUD1** |  |  |  |  |  |  |  | 1 |  |  |  |  |  |  |  |  |
| **GOT1** |  |  |  |  | 0.66 |  | 0.03 |  |  | 1 |  |  |  |  |  |  |
| **GOT2** |  |  |  |  |  |  |  |  |  |  |  | 1 |  |  |  |  |
| **GPT2** |  |  |  |  |  |  |  |  |  |  |  | 0.78 | 0.00 | 1 |  |  |
|  |  | **GBM** | | | | | | | | | | | | | | |
|  | **GLSiso1** | | **GLSiso2** | |  | **GLS2** | | **GLUD1** | | **GOT1** | | **GOT2** | | **GPT2** | |  |
|  | **r** | **p** | **r** | **p** | **r** |  | **p** | **r** | **p** | **r** | **p** | **r** | **p** | **r** | **p** |  |
| **GLSiso1** | 1 |  |  |  |  |  |  |  |  |  |  |  |  |  |  |  |
| **GLSiso2** | 0.64 | 0.00 | 1 |  |  |  |  |  |  |  |  |  |  |  |  |  |
| **GLS2** | 0.43 | 0.00 | 0.1 | 0.04 | 1 |  |  |  |  |  |  |  |  |  |  |  |
| **GLUD1** | 0.69 | 0.00 | 0.49 | 0.00 |  |  |  | 1 |  |  |  |  |  |  |  |  |
| **GOT1** | 0.68 | 0.00 | 0.43 | 0.00 | 0.41 |  | 0.00 | 0.7 | 0.00 | 1 |  |  |  |  |  |  |
| **GOT2** | 0.65 | 0.00 | 0.56 | 0.00 |  |  |  | 0.78 | 0.00 | 0.73 | 0.00 | 1 |  |  |  |  |
| **GPT2** | 0.67 | 0.00 | 0.7 | 0.00 | 0.27 |  | 0.00 | 0.61 | 0.00 | 0.55 | 0.00 | 0.67 | 0.00 | 1 |  |  |

(NN- Non neoplastic, AGI: pilocytic astrocytoma, AGII: low grade astrocytoma, AGIII: anaplastic astrocytoma and GBM (glioblastoma). The values indicate the levels of correlations (r) ranging from -1 (inverse or weak correlation) to 1 (strong correlation) by Spearman´s correlation test (only the correlations with p < 0.05 were plotted).
