# Supplementary material for: Glutaminolysis dynamics during astrocytoma progression correlates with tumor aggressiveness
Source: Cancer Metab. 2021 Apr 28;9:18. doi: 10.1186/s40170-021-00255-8 (PMC8082835; doi:10.1186/s40170-021-00255-8)
Supplement: Supplementary file 5 — Additional file 5: Supplemental Table S2. Correlations values of the glutaminolysis and GSH pathway genes in the subtypes of GBM. [file 40170_2021_255_MOESM5_ESM.docx]

**Supplemental Table 2. Correlations values of the glutaminolysis and GSH pathway genes in all subtypes of GBM.**

|  |  |  |  |  |  |  |  |  |  |  |  |  |  |  |  |  |  |  |  |  |  |  |  |  |  |  |
| --- | --- | --- | --- | --- | --- | --- | --- | --- | --- | --- | --- | --- | --- | --- | --- | --- | --- | --- | --- | --- | --- | --- | --- | --- | --- | --- |
|  | **PN** | | | | | | | | | | | | | | | | | | | | | | | | | |
|  | **GLS** | | **GLS2** | | **GLUD1** | | **GOT1** | | **GOT2** | | **GPT2** | | **GCLM** | | **GGCT** | | **GSR** | | **GSTM4** | | **GSTO1** | | **MGST1** | | **MGST2** | |
|  | **r** | **p** | **r** | **p** | **r** | **p** | **r** | **p** | **r** | **p** | **r** | **p** | **r** | **p** | **r** | **p** | **r** | **p** | **r** | **p** | **r** | **p** | **r** | **p** | **r** | **p** |
| **GLS** | 1 |  |  |  |  |  |  |  |  |  |  |  |  |  |  |  |  |  |  |  |  |  |  |  |  |  |
| **GLS2** |  |  | 1 |  |  |  |  |  |  |  |  |  |  |  |  |  |  |  |  |  |  |  |  |  |  |  |
| **GLUD1** |  |  |  |  | 1 |  |  |  |  |  |  |  |  |  |  |  |  |  |  |  |  |  |  |  |  |  |
| **GOT1** |  |  |  |  | 0.39 | 0.02 | 1 |  |  |  |  |  |  |  |  |  |  |  |  |  |  |  |  |  |  |  |
| **GOT2** |  |  |  |  |  |  |  |  | 1 |  |  |  |  |  |  |  |  |  |  |  |  |  |  |  |  |  |
| **GPT2** |  |  |  |  | 0.27 | 0.00 |  |  |  |  | 1 |  |  |  |  |  |  |  |  |  |  |  |  |  |  |  |
| **GCLM** |  |  |  |  |  |  | 0.47 | 0.03 | 0.35 | 0.05 |  |  | 1 |  |  |  |  |  |  |  |  |  |  |  |  |  |
| **GGCT** |  |  |  |  |  |  |  |  |  |  |  |  |  |  | 1 |  |  |  |  |  |  |  |  |  |  |  |
| **GSR** |  |  |  |  |  |  |  |  |  |  |  |  |  |  | 0.13 | 0.05 | 1 |  |  |  |  |  |  |  |  |  |
| **GSTM4** |  |  | 0.49 | 0.0296 | 0.49 | 0.01 |  |  |  |  |  |  | 0.53 | 0.02 |  |  | 0.51 | 0.00 | 1 |  |  |  |  |  |  |  |
| **GSTO1** |  |  |  |  |  |  | 0.44 | 0.01 |  |  |  |  |  |  |  |  | 0.57 | 0.00 |  |  | 1 |  |  |  |  |  |
| **MGST1** |  |  |  |  |  |  |  |  |  |  |  |  | 0.34 | 0.03 |  |  |  |  |  |  |  |  | 1 |  |  |  |
| **MGST2** |  |  |  |  |  |  |  |  |  |  |  |  |  |  |  |  | 0.37 | 0.01 | 0.51 | 0.00 | 0.57 | 0.01 |  |  | 1 |  |
|  |  |  |  |  |  |  |  |  |  |  |  |  |  |  |  |  |  |  |  |  |  |  |  |  |  |  |
|  | **CS** | | | | | | | | | | | | | | | | | | | | | | | | | |
|  | **GLS** | | **GLS2** | | **GLUD1** | | **GOT1** | | **GOT2** | | **GPT2** | | **GCLM** | | **GGCT** | | **GSR** | | **GSTM4** | | **GSTO1** | | **MGST1** | | **MGST2** | |
|  | **r** | **p** | **r** | **p** | **r** | **p** | **r** | **p** | **r** | **p** | **r** | **p** | **r** | **p** | **r** | **p** | **r** | **p** | **r** | **p** | **r** | **p** | **r** | **p** | **r** | **p** |
| **GLS** | 1 |  |  |  |  |  |  |  |  |  |  |  |  |  |  |  |  |  |  |  |  |  |  |  |  |  |
| **GLS2** |  |  | 1 |  |  |  |  |  |  |  |  |  |  |  |  |  |  |  |  |  |  |  |  |  |  |  |
| **GLUD1** |  |  |  |  | 1 |  |  |  |  |  |  |  |  |  |  |  |  |  |  |  |  |  |  |  |  |  |
| **GOT1** | 0.4 | 0.00 | 0.06 | 0.02 | 0.46 | 0.04 | 1 |  |  |  |  |  |  |  |  |  |  |  |  |  |  |  |  |  |  |  |
| **GOT2** | 0.34 | 0.00 |  |  | 0.43 | 0.03 | 0.37 | 0.01 | 1 |  |  |  |  |  |  |  |  |  |  |  |  |  |  |  |  |  |
| **GPT2** |  |  |  |  |  |  |  |  |  |  | 1 |  |  |  |  |  |  |  |  |  |  |  |  |  |  |  |
| **GCLM** |  |  |  |  |  |  |  |  |  |  |  |  | 1 |  |  |  |  |  |  |  |  |  |  |  |  |  |
| **GGCT** |  |  |  |  |  |  |  |  |  |  |  |  |  |  | 1 |  |  |  |  |  |  |  |  |  |  |  |
| **GSR** |  |  | 0.51 | 0.00 |  |  |  |  | 0.33 | 0.01 |  |  |  |  |  |  | 1 |  |  |  |  |  |  |  |  |  |
| **GSTM4** |  |  |  |  |  |  |  |  |  |  |  |  |  |  |  |  | 0.41 | 0.00 | 1 |  |  |  |  |  |  |  |
| **GSTO1** | 0.24 | 0.01 | 0.61 | 0.01 |  |  |  |  | 0.44 | 0.00 |  |  |  |  |  |  | 0.67 | 0.00 |  |  | 1 |  |  |  |  |  |
| **MGST1** |  |  |  |  |  |  |  |  |  |  |  |  | 0.42 | 0.00 |  |  |  |  |  |  |  |  | 1 |  |  |  |
| **MGST2** |  |  | 0.33 | 0.04 |  |  |  |  |  |  |  |  |  |  | 0.42 | 0.00 | 0.47 | 0.00 |  |  | 0.57 | 0.00 |  |  | 1 |  |
|  |  |  |  |  |  |  |  |  |  |  |  |  |  |  |  |  |  |  |  |  |  |  |  |  |  |  |
|  | **MS** | | | | | | | | | | | | | | | | | | | | | | | | | |
|  | **GLS** | | **GLS2** | | **GLUD1** | | **GOT1** | | **GOT2** | | **GPT2** | | **GCLM** | | **GGCT** | | **GSR** | | **GSTM4** | | **GSTO1** | | **MGST1** | | **MGST2** | |
|  | **r** | **p** | **r** | **p** | **r** | **p** | **r** | **p** | **r** | **p** | **r** | **p** | **r** | **p** | **r** | **p** | **r** | **p** | **r** | **p** | **r** | **p** | **r** | **p** | **r** | **p** |
| **GLS** | 1 |  |  |  |  |  |  |  |  |  |  |  |  |  |  |  |  |  |  |  |  |  |  |  |  |  |
| **GLS2** |  |  | 1 |  |  |  |  |  |  |  |  |  |  |  |  |  |  |  |  |  |  |  |  |  |  |  |
| **GLUD1** | 0.31 | 0.03 |  |  | 1 |  |  |  |  |  |  |  |  |  |  |  |  |  |  |  |  |  |  |  |  |  |
| **GOT1** |  |  |  |  |  |  | 1 |  |  |  |  |  |  |  |  |  |  |  |  |  |  |  |  |  |  |  |
| **GOT2** |  |  |  |  |  |  | 0.3 | 0.00 | 1 |  |  |  |  |  |  |  |  |  |  |  |  |  |  |  |  |  |
| **GPT2** | 0.54 | 0.00 |  |  | 0.48 | 0.00 |  |  | 0.41 | 0.02 | 1 |  |  |  |  |  |  |  |  |  |  |  |  |  |  |  |
| **GCLM** | 0.11 | 0.00 |  |  |  |  |  |  |  |  |  |  | 1 |  |  |  |  |  |  |  |  |  |  |  |  |  |
| **GGCT** |  |  |  |  |  |  |  |  |  |  |  |  |  |  | 1 |  |  |  |  |  |  |  |  |  |  |  |
| **GSR** | 0.15 | 0.01 |  |  |  |  | 0.27 | 0.01 |  |  | 0.36 | 0.03 | 0.06 | 0.00 | 0.33 | 0.01 | 1 |  |  |  |  |  |  |  |  |  |
| **GSTM4** |  |  |  |  |  |  | 0.39 | 0.00 |  |  |  |  |  |  |  |  |  |  | 1 |  |  |  |  |  |  |  |
| **GSTO1** |  |  |  |  |  |  |  |  |  |  | 0.49 | 0.00 | 0.27 | 0.04 |  |  |  |  | 0.4 | 0.01 | 1 |  |  |  |  |  |
| **MGST1** |  |  |  |  |  |  |  |  |  |  |  |  | 0.51 | 0.00 | 0.1 | 0.03 |  |  |  |  | 0.37 | 0.00 | 1 |  |  |  |
| **MGST2** |  |  |  |  |  |  |  |  |  |  | 0.32 | 0.01 |  |  |  |  |  |  | 0.67 | 0.00 | 0.58 | 0.00 | 0.34 | 0.01 | 1 |  |
|  |  |  |  |  |  |  |  |  |  |  |  |  |  |  |  |  |  |  |  |  |  |  |  |  |  |  |
|  | **GBM** | | | | | | | | | | | | | | | | | | | | | | | | | |
|  | **GLS** | | **GLS2** | | **GLUD1** | | **GOT1** | | **GOT2** | | **GPT2** | | **GCLM** | | **GGCT** | | **GSR** | | **GSTM4** | | **GSTO1** | | **MGST1** | | **MGST2** | |
|  | **r** | **p** | **r** | **p** | **r** | **p** | **r** | **p** | **r** | **p** | **r** | **p** | **r** | **p** | **r** | **p** | **r** | **p** | **r** | **p** | **r** | **p** | **r** | **p** | **r** | **p** |
| **GLS** | 1 |  |  |  |  |  |  |  |  |  |  |  |  |  |  |  |  |  |  |  |  |  |  |  |  |  |
| **GLS2** |  |  | 1 |  |  |  |  |  |  |  |  |  |  |  |  |  |  |  |  |  |  |  |  |  |  |  |
| **GLUD1** |  |  |  |  | 1 |  |  |  |  |  |  |  |  |  |  |  |  |  |  |  |  |  |  |  |  |  |
| **GOT1** | 0.26 | 0.03 | 0.1 | 0.04 | 0.28 | 0.01 | 1 |  |  |  |  |  |  |  |  |  |  |  |  |  |  |  |  |  |  |  |
| **GOT2** |  |  |  |  |  |  | 0.33 | 0.00 | 1 |  |  |  |  |  |  |  |  |  |  |  |  |  |  |  |  |  |
| **GPT2** | -0.3 | 0.00 |  |  | 0.34 | 0.00 |  |  |  |  | 1 |  |  |  |  |  |  |  |  |  |  |  |  |  |  |  |
| **GCLM** | 0.07 | 0.00 |  |  |  |  |  |  |  |  | 0.27 | 0.00 | 1 |  |  |  |  |  |  |  |  |  |  |  |  |  |
| **GGCT** |  |  | 0.24 | 0.01 | 0.17 | 0.05 |  |  |  |  | -0.4 | 0.00 | 0.35 | 0.01 | 1 |  |  |  |  |  |  |  |  |  |  |  |
| **GSR** | 0.05 | 0.00 | 0.36 | 0.00 |  |  | 0.15 | 0.01 |  |  | 0.36 | 0.00 | 0.29 | 0.00 | 0.41 | 0.00 | 1 |  |  |  |  |  |  |  |  |  |
| **GSTM4** |  |  |  |  |  |  | 0.08 | 0.04 |  |  | 0.31 | 0.00 |  |  | 0.38 | 0.00 | 0.42 | 0.00 | 1 |  |  |  |  |  |  |  |
| **GSTO1** | 0.21 | 0.00 | 0.33 | 0.01 |  |  | 0.33 | 0.01 |  |  | 0.49 | 0.00 | 0.35 | 0.00 | 0.35 | 0.01 | 0.55 | 0.00 | 0.36 | 0.00 | 1 |  |  |  |  |  |
| **MGST1** |  |  |  |  |  |  |  |  | -0.2 | 0.02 | 0.22 | 0.01 | 0.59 | 0.00 | 0.28 | 0.00 | 0.23 | 0.00 | 0.26 | 0.00 | 0.36 | 0.00 | 1 |  |  |  |
| **MGST2** |  |  |  |  |  |  |  |  |  |  | 0.38 | 0.00 | 0.3 | 0.03 | 0.47 | 0.00 | 0.33 | 0.01 | 0.58 | 0.00 | 0.6 | 0.00 | 0.36 | 0.00 | 1 |  |

(PN) proneural (CS) classical, (MS) mesenchymal, and total GBM. The values indicate the levels of correlations (r) ranging from -1 (inverse or weak correlation) to 1 (strong correlation) by Spearman´s correlation test (only the correlations with p < 0.05 were plotted).
