# Supplementary material for: Glutaminolysis dynamics during astrocytoma progression correlates with tumor aggressiveness
Source: Cancer Metab. 2021 Apr 28;9:18. doi: 10.1186/s40170-021-00255-8 (PMC8082835; doi:10.1186/s40170-021-00255-8)
Supplement: Supplementary file 6 — Additional file 6: Supplemental Table S3. Correlations values of the glutaminolysis and GSH pathway genes in AGII and AGIII with IDH1 wild type (IDH1wt) and mutated IDH1 (IDH1mut). [file 40170_2021_255_MOESM6_ESM.docx]

**Supplemental Table 3. Correlations values of the glutaminolysis and GSH pathway genes in AGII and AGIII *IDH1* wild type (*IDH1*^wt^) and mutated *IDH1* (*IDH1*^mut^).**

|  | **AGII*IDH1*^wt^** | | | | | | | | | | | | | | | | | | | | | | | | | |
| --- | --- | --- | --- | --- | --- | --- | --- | --- | --- | --- | --- | --- | --- | --- | --- | --- | --- | --- | --- | --- | --- | --- | --- | --- | --- | --- |
|  | **GLS** | | **GLS2** | | **GLUD1** | | **GOT1** | | **GOT2** | | **GPT2** | | **GCLM** | | **GGCT** | | **GSR** | | **GSTM4** | | **GSTO1** | | **MGST1** | | **MGST2** | |
|  | **r** | **p** | **r** | **p** | **r** | **p** | **r** | **p** | **r** | **p** | **r** | **p** | **r** | **p** | **r** | **p** | **r** | **p** | **r** | **p** | **r** | **p** | **r** | **p** | **r** | **p** |
| **GLS** | 1 |  |  |  |  |  |  |  |  |  |  |  |  |  |  |  |  |  |  |  |  |  |  |  |  |  |
| **GLS2** | 0.93 | 0.000 | 1 |  |  |  |  |  |  |  |  |  |  |  |  |  |  |  |  |  |  |  |  |  |  |  |
| **GLUD1** |  |  |  |  | 1 |  |  |  |  |  |  |  |  |  |  |  |  |  |  |  |  |  |  |  |  |  |
| **GOT1** | 0.68 | 0.000 | 0.63 | 0.000 |  |  | 1 |  |  |  |  |  |  |  |  |  |  |  |  |  |  |  |  |  |  |  |
| **GOT2** | 0.15 | 0.013 |  |  |  |  | 0.38 | 0.016 | 1 |  |  |  |  |  |  |  |  |  |  |  |  |  |  |  |  |  |
| **GPT2** |  |  |  |  |  |  |  |  |  |  | 1 |  |  |  |  |  |  |  |  |  |  |  |  |  |  |  |
| **GCLM** |  |  |  |  |  |  |  |  |  |  |  |  | 1 |  |  |  |  |  |  |  |  |  |  |  |  |  |
| **GGCT** |  |  |  |  |  |  |  |  |  |  |  |  |  |  | 1 |  |  |  |  |  |  |  |  |  |  |  |
| **GSR** |  |  |  |  |  |  |  |  |  |  |  |  |  |  |  |  | 1 |  |  |  |  |  |  |  |  |  |
| **GSTM4** |  |  |  |  |  |  |  |  |  |  |  |  |  |  |  |  |  |  | 1 |  |  |  |  |  |  |  |
| **GSTO1** |  |  |  |  |  |  |  |  |  |  |  |  |  |  |  |  |  |  | 0.68 | 0.005 | 1 |  |  |  |  |  |
| **MGST1** |  |  |  |  |  |  |  |  |  |  |  |  |  |  |  |  |  |  |  |  |  |  | 1 |  |  |  |
| **MGST2** |  |  |  |  |  |  |  |  |  |  |  |  |  |  |  |  |  |  | 0.38 | 0.018 | 0.28 | 0.002 |  |  | 1 |  |
|  | **AGIII*IDH1*^wt^** | | | | | | | | | | | | | | | | | | | | | | | | | |
|  | **GLS** | | **GLS2** | | **GLUD1** | | **GOT1** | | **GOT2** | | **GPT2** | | **GCLM** | | **GGCT** | | **GSR** | | **GSTM4** | | **GSTO1** | | **MGST1** | | **MGST2** | |
|  | **r** | **p** | **r** | **p** | **r** | **p** | **r** | **p** | **r** | **p** | **r** | **p** | **r** | **p** | **r** | **p** | **r** | **p** | **r** | **p** | **r** | **p** | **r** | **p** | **r** | **p** |
| **GLS** | 1 |  |  |  |  |  |  |  |  |  |  |  |  |  |  |  |  |  |  |  |  |  |  |  |  |  |
| **GLS2** | 0.38 | 0.000 | 1 |  |  |  |  |  |  |  |  |  |  |  |  |  |  |  |  |  |  |  |  |  |  |  |
| **GLUD1** | 0.05 | 0.036 |  |  | 1 |  |  |  |  |  |  |  |  |  |  |  |  |  |  |  |  |  |  |  |  |  |
| **GOT1** | 0.38 | 0.000 | 0.48 | 0.000 | 0.13 | 0.026 | 1 |  |  |  |  |  |  |  |  |  |  |  |  |  |  |  |  |  |  |  |
| **GOT2** |  |  |  |  |  |  | 0.49 | 0.000 | 1 |  |  |  |  |  |  |  |  |  |  |  |  |  |  |  |  |  |
| **GPT2** |  |  |  |  | 0.43 | 0.014 |  |  |  |  | 1 |  |  |  |  |  |  |  |  |  |  |  |  |  |  |  |
| **GCLM** |  |  |  |  |  |  |  |  | 0.32 | 0.007 |  |  | 1 |  |  |  |  |  |  |  |  |  |  |  |  |  |
| **GGCT** |  |  |  |  |  |  |  |  |  |  |  |  |  |  | 1 |  |  |  |  |  |  |  |  |  |  |  |
| **GSR** |  |  |  |  |  |  |  |  | 0.32 | 0.021 |  |  | 0.5 | 0.008 |  |  | 1 |  |  |  |  |  |  |  |  |  |
| **GSTM4** |  |  |  |  | 0.19 | 0.024 |  |  |  |  |  |  |  |  |  |  |  |  | 1 |  |  |  |  |  |  |  |
| **GSTO1** |  |  |  |  |  |  |  |  |  |  |  |  |  |  |  |  |  |  | 0.66 | 0.000 | 1 |  |  |  |  |  |
| **MGST1** |  |  |  |  |  |  |  |  |  |  |  |  | 0.41 | 0.000 |  |  |  |  |  |  | 0.32 | 0.019 | 1 |  |  |  |
| **MGST2** |  |  | 0.48 | 0.003 |  |  |  |  |  |  |  |  |  |  |  |  |  |  | 0.53 | 0.012 | 0.59 | 0.000 |  |  | 1 |  |
|  |  |  |  |  |  |  |  |  |  |  |  |  |  |  |  |  |  |  |  |  |  |  |  |  |  |  |
|  | **AGII*IDH1*^mut^** | | | | | | | | | | | | | | | | | | | | | | | | | |
|  | **GLS** | | **GLS2** | | **GLUD1** | | **GOT1** | | **GOT2** | | **GPT2** | | **GCLM** | | **GGCT** | | **GSR** | | **GSTM4** | | **GSTO1** | | **MGST1** | | **MGST2** | |
|  | **r** | **p** | **r** | **p** | **r** | **p** | **r** | **p** | **r** | **p** | **r** | **p** | **r** | **p** | **r** | **p** | **r** | **p** | **r** | **p** | **r** | **p** | **r** | **p** | **r** | **p** |
| **GLS** | 1 |  |  |  |  |  |  |  |  |  |  |  |  |  |  |  |  |  |  |  |  |  |  |  |  |  |
| **GLS2** | 0.63 | 0.000 | 1 |  |  |  |  |  |  |  |  |  |  |  |  |  |  |  |  |  |  |  |  |  |  |  |
| **GLUD1** |  |  |  |  | 1 |  |  |  |  |  |  |  |  |  |  |  |  |  |  |  |  |  |  |  |  |  |
| **GOT1** | 0.65 | 0.000 | 0.7 | 0.000 |  |  | 1 |  |  |  |  |  |  |  |  |  |  |  |  |  |  |  |  |  |  |  |
| **GOT2** | 0.66 | 0.000 | 0.41 | 0.000 |  |  | 0.68 | 0.000 | 1 |  |  |  |  |  |  |  |  |  |  |  |  |  |  |  |  |  |
| **GPT2** |  |  |  |  | 0.44 | 0.001 |  |  |  |  | 1 |  |  |  |  |  |  |  |  |  |  |  |  |  |  |  |
| **GCLM** | 0.31 | 0.019 |  |  | 0.32 | 0.044 | 0.33 | 0.037 | 0.43 | 0.014 |  |  | 1 |  |  |  |  |  |  |  |  |  |  |  |  |  |
| **GGCT** |  |  | 0.1 | 0.047 |  |  | 0.39 | 0.011 |  |  |  |  |  |  | 1 |  |  |  |  |  |  |  |  |  |  |  |
| **GSR** |  |  |  |  |  |  |  |  | 0.39 | 0.023 |  |  | 0.44 | 0.000 | 0.33 | 0.038 | 1 |  |  |  |  |  |  |  |  |  |
| **GSTM4** |  |  |  |  | 0.33 | 0.012 |  |  |  |  |  |  |  |  | 0.48 | 0.003 |  |  | 1 |  |  |  |  |  |  |  |
| **GSTO1** |  |  |  |  |  |  | 0.5 | 0.005 |  |  |  |  |  |  | 0.69 | 0.000 | 0.37 | 0.023 |  |  | 1 |  |  |  |  |  |
| **MGST1** |  |  |  |  |  |  |  |  |  |  | 0.47 | 0.002 | 0.52 | 0.013 |  |  | 0.24 | 0.009 |  |  | 0.49 | 0.000 | 1 |  |  |  |
| **MGST2** |  |  | 0.29 | 0.044 |  |  |  |  |  |  |  |  |  |  | 0.3 | 0.037 | 0.4 | 0.000 |  |  | 0.43 | 0.003 | 0.22 | 0.020 | 1 |  |
|  | **AGIII*IDH1*^mut^** | | | | | | | | | | | | | | | | | | | | | | | | | |
|  | **GLS** | | **GLS2** | | **GLUD1** | | **GOT1** | | **GOT2** | | **GPT2** | | **GCLM** | | **GGCT** | | **GSR** | | **GSTM4** | | **GSTO1** | | **MGST1** | | **MGST2** | |
|  | **r** | **p** | **r** | **p** | **r** | **p** | **r** | **p** | **r** | **p** | **r** | **p** | **r** | **p** | **r** | **p** | **r** | **p** | **r** | **p** | **r** | **p** | **r** | **p** | **r** | **p** |
| **GLS** | 1 |  |  |  |  |  |  |  |  |  |  |  |  |  |  |  |  |  |  |  |  |  |  |  |  |  |
| **GLS2** | 0.55 | 0.000 | 1 |  |  |  |  |  |  |  |  |  |  |  |  |  |  |  |  |  |  |  |  |  |  |  |
| **GLUD1** |  |  |  |  | 1 |  |  |  |  |  |  |  |  |  |  |  |  |  |  |  |  |  |  |  |  |  |
| **GOT1** | 0.49 | 0.000 | 0.48 | 0.000 |  |  | 1 |  |  |  |  |  |  |  |  |  |  |  |  |  |  |  |  |  |  |  |
| **GOT2** | 0.22 | 0.001 | 0.11 | 0.004 |  |  | 0.36 | 0.000 | 1 |  |  |  |  |  |  |  |  |  |  |  |  |  |  |  |  |  |
| **GPT2** |  |  |  |  | 0.69 | 0.000 |  |  |  |  | 1 |  |  |  |  |  |  |  |  |  |  |  |  |  |  |  |
| **GCLM** |  |  |  |  | 0.15 | 0.025 |  |  |  |  |  |  | 1 |  |  |  |  |  |  |  |  |  |  |  |  |  |
| **GGCT** |  |  |  |  |  |  |  |  |  |  |  |  |  |  | 1 |  |  |  |  |  |  |  |  |  |  |  |
| **GSR** |  |  |  |  | 0.15 | 0.023 |  |  |  |  | 0.25 | 0.001 | 0.29 | 0.030 |  |  | 1 |  |  |  |  |  |  |  |  |  |
| **GSTM4** | 0.31 | 0.021 |  |  |  |  |  |  |  |  |  |  |  |  |  |  |  |  | 1 |  |  |  |  |  |  |  |
| **GSTO1** |  |  |  |  | 0.16 | 0.032 |  |  | 0.16 | 0.049 |  |  |  |  | 0.47 | 0.000 | 0.23 | 0.009 | 0.29 | 0.019 | 1 |  |  |  |  |  |
| **MGST1** |  |  |  |  |  |  |  |  |  |  |  |  | 0.52 | 0.001 |  |  |  |  | 0.37 | 0.004 | 0.46 | 0.003 | 1 |  |  |  |
| **MGST2** |  |  | 0.28 | 0.017 | 0.18 | 0.021 |  |  | 0.28 | 0.028 | -0.2 | 0.031 | 0.29 | 0.044 |  |  | 0.3 | 0.000 | 0.44 | 0.000 | 0.43 | 0.000 | 0.38 | 0.000 | 1 |  |

The values indicate the levels of correlations (r) ranging from -1 (inverse or weak correlation) to 1 (strong correlation) by Spearman´s correlation test (only the correlations with p < 0.05 were plotted).
